# Composition of the Solvation Shell of the Selected Cyclic Ethers (1,4-Dioxane, 12-Crown-4, 15-Crown-5 and 18-Crown-6) in a Mixture of Formamide with Water at Four Temperatures

**DOI:** 10.3390/molecules28052169

**Published:** 2023-02-26

**Authors:** Małgorzata Jóźwiak, Monika A. Trzmielak, Michał Wasiak, Katarzyna Łudzik-Dychto

**Affiliations:** Department of Physical Chemistry, Faculty of Chemistry, University of Lodz, Pomorska 165, 90-236 Lodz, Poland

**Keywords:** 15-crown-5 ether, 18-crown-6 ether, enthalpy of solution, preferential solvation, formamide-water mixtures, composition of the solvation shell

## Abstract

The solution enthalpy of 15-crown-5 and 18-crown-6 ethers in the mixture of formamide (F) and water (W) was measured at four temperatures: 293.15 K, 298.15 K, 303.15 K, 308.15 K. The standard molar enthalpy of solution, $\Delta_{\mathrm{sol}}H^{o}$, depends on the size of cyclic ethers molecules and the temperature. With increasing temperature, the values of $\Delta_{\mathrm{sol}}H^{o}$ become less negative. The values of the standard partial molar heat capacity $C_{p,2}^{o}$ of cyclic ethers at 298.15 K have been calculated. The $C_{p,2}^{o}=f(x_{W})$ curve shape indicates the hydrophobic hydration process of cyclic ethers in the range of a high-water content in the mixture with formamide. The enthalpic effect of preferential solvation of cyclic ethers was calculated and the effect of temperature on the preferential solvation process was discussed. The process of complex formation between 18C6 molecules and formamide molecules is observed. The cyclic ethers molecules are preferentially solvated by formamide molecules. The mole fraction of formamide in the solvation sphere of cyclic ethers has been calculated.

## 1. Introduction

The solvation process has a large impact on the solute–solute intermolecular interactions [1,2,3] and thus also on the course of chemical reactions, i.e., in the reaction mechanism. The knowledge of the thermodynamics of the solvation process, including preferential solvation (PS) or hydrophobic hydration (HH), plays a very important role in the selection of the solvent for the chemical reaction [4]. In the mixed aqueous–organic or organic–organic solvents it is likely that a preferential solvation process will occur. Moreover, in water-rich mixed aqueous–organic solvents a substance which shows a hydrophobic character, can be hydrophobically hydrated. The preferential solvation process is defined as a difference in the solvent components between the solvation shell and the bulk solvent [5,6,7]. The process of the preferential solvation occurring in the mixed, often multi-component solvents has been deeply investigated by many researchers [8,9,10,11,12,13,14,15]. 

Crown ethers belong to the group of compounds characterized by a hydrophobic-hydrophilic character. In a pure water they are hydrophobically hydrated [16,17] and in mixed water–organic solvents they may be preferentially solvated by one of the components of the mixture [18]. Cyclic ethers have numerous uses including biology, medicine, or the pharmaceutical industry [19,20,21,22,23,24,25]. Crown ethers, although the most popular at the end of the 20th century, are still popular among scientists due to their wide application in various fields.

This publication is a continuation of our research on the influence of the type of solvent and the temperature on the process of preferential solvation of crown ethers [16,17,26,27]. 15-crown-5 (15C5), 18-crown-6 (18C6) and a formamide-water (F + W) mixture were selected for the investigations. The hydrophilic molecules of formamide (F) are capable of forming hydrogen bonds [28]. Formamide as a solvent is used in chemistry for dissolution of many organic substances [29] as well as in biology for different study [30] and industry [31].

## 2. Results and Discussion

### 2.1. Enthalpy of Solution and Heat Capacity

**Figure 1:**
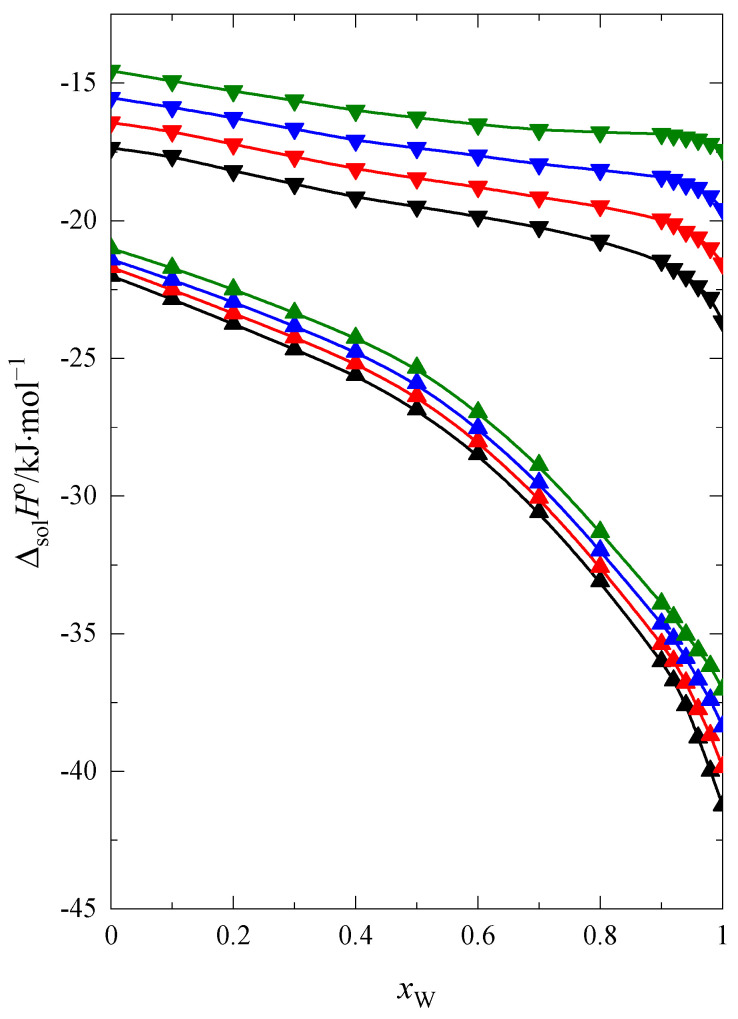
The standard solution enthalpy of 15C5 (▲) and 18C6 (▼) in the F + W mixtures as a function of molar fraction of water at: 293.15 K (black line), 298.15 K (red line), 303.15 K (blue line), 308.15 K (green line).

The standard solution enthalpy of 15C5 and 18C6 at four temperatures within the range: (293.15–308.15) K as a function of the mole fraction of water, *x*_w_ in the F + W mixture are presented in Figure 1. 

As seen in Figure 1 the enthalpy of solution increases (the process of solution becomes less exothermic) with increasing of temperature. Within the whole range of the water mole fraction the course of curves $\Delta_{\mathrm{sol}}H_{}^{o}=f(x_{W})$ is monotonic and decreases with an increase of the *x*_W_ in the mixture. In the case of 18C6, the changes of this function are smaller. This indicates some differences in interactions between 15C5 and 18C6 molecules with mixed solvent molecules. 

The data of the standard molar enthalpy of solution of 15C5 and 18C6 obtained in the presented paper at four temperatures and analogous data for 1,4-dioxane and 12-crown-4 (12C4) presented in our previous paper [16] allow us to calculate the standard molar heat of capacity of solution of cyclic ethers using Equation (1).

(1) $$\Delta_{\mathrm{sol}}C_{p}^{o}={\left(\partial \Delta_{\mathrm{sol}}H^{o}/\partial T\right)}_{p}$$

Then, the standard partial molar heat capacity ($C_{p,2}^{o}$) of the cyclic ethers was been calculated using Equation (2):

(2) $$C_{p,2}^{o}=\Delta_{\mathrm{sol}}C_{p}^{o}+C_{p}^{*}$$

where: $C_{p}^{*}$ is the molar heat capacity of pure cyclic ethers. 

**Figure 2:**
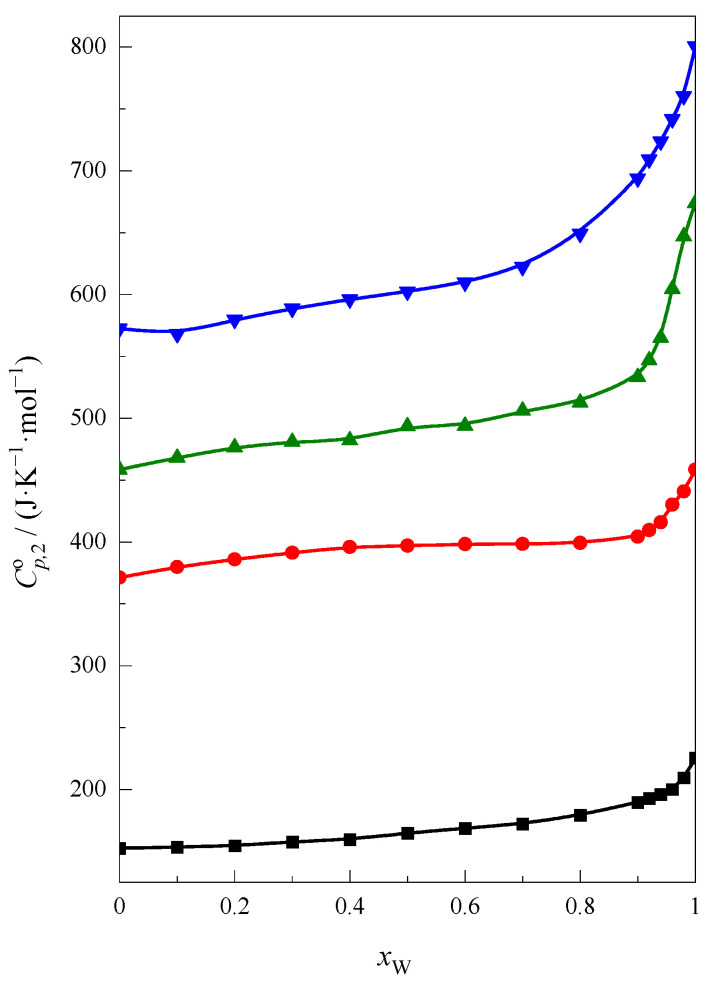
The standard partial molar heat capacity of cyclic ethers: ■, 1,4-dioxane, ●, 12C4, ▲, 15C5, ▼, 18C6, as a function of *x*_W_ in the F + W mixture at 298.15 K.

The molar heat capacity of pure compounds was taken from the literature [32,33] for 1,4-dioxane and crown ethers, respectively. The values of the standard partial molar heat capacity ($C_{p,2}^{o}$) of cyclic ethers in water 225.4 J∙K^−1^∙mol^−1^ for 1,4-dioxane, 458.5 J∙K^−1^∙mol^−1^ for 12C4, 674.0 J∙K^−1^∙mol^−1^ for 15C5 and 800.5 J∙K^−1^∙mol^−1^ for 18C6 are in agreement with the literature data [33,34] for 1,4-dioxane and crown ethers, respectively. The results obtained at 298.15 K are presented in Figure 2. 

As seen in Figure 2 the courses of curves are similar for 1,4-dioxane, 12C4 and 15C5. These shapes can be divided into two areas. First, with small and medium water content (*x*_W_ < 0.9) and second, with a high-water content in the F + W mixture (*x*_W_ ≥ 0.9). In the first area a linear, very small increase in the standard partial molar heat capacity of cyclic ethers with increasing of a water content in the mixture is observed. For 18C6, the shape of this curve is slightly different from the others in the range of 0.1 > *x*_W_ > 0.7. In the area of high formamide content (*x*_W_ < 0.1), the small decrease of the standard partial molar heat capacity with the increasing water is observed. It suggests that 18C6 interacts in a specific way with formamide. In the literature [35,36] the crystal structure of the 1:2 complex of 18C6 molecule with formamide molecules is described. There are reports on the formation of complexes of 18C6 in solution with solvents such as: nitromethane, acetonitrile, chloroform or acetone [37]. Analyzing the distribution of the charges on hydrogen atoms in formamide, acetonitrile and acetone [18], it can be seen that in the case of formamide the value of this charge is the highest. Therefore, it can be assumed that such complexes between the 18C6 and formamide molecules may form in the solution too.

In the second area, an abrupt increase in the value of the standard partial molar heat capacity of cyclic ethers with increasing of water content can be observed. This is probably related to the hydrophobic hydration process of cyclic ethers, which enthalpic effect is the smallest for 1,4-dioxane and the highest for 18C6. Additionally in the case of 18C6 this increase of the function $C_{p,2}^{o}=f(x_{W})$ starts from *x*_W_ ≈ 0.7 but in case of other ethers, from *x*_W_ ≈ 0.9. This may indicate a different behavior of 18C6 molecules from other cyclic ethers also in the water-rich region.

**Figure 3:**
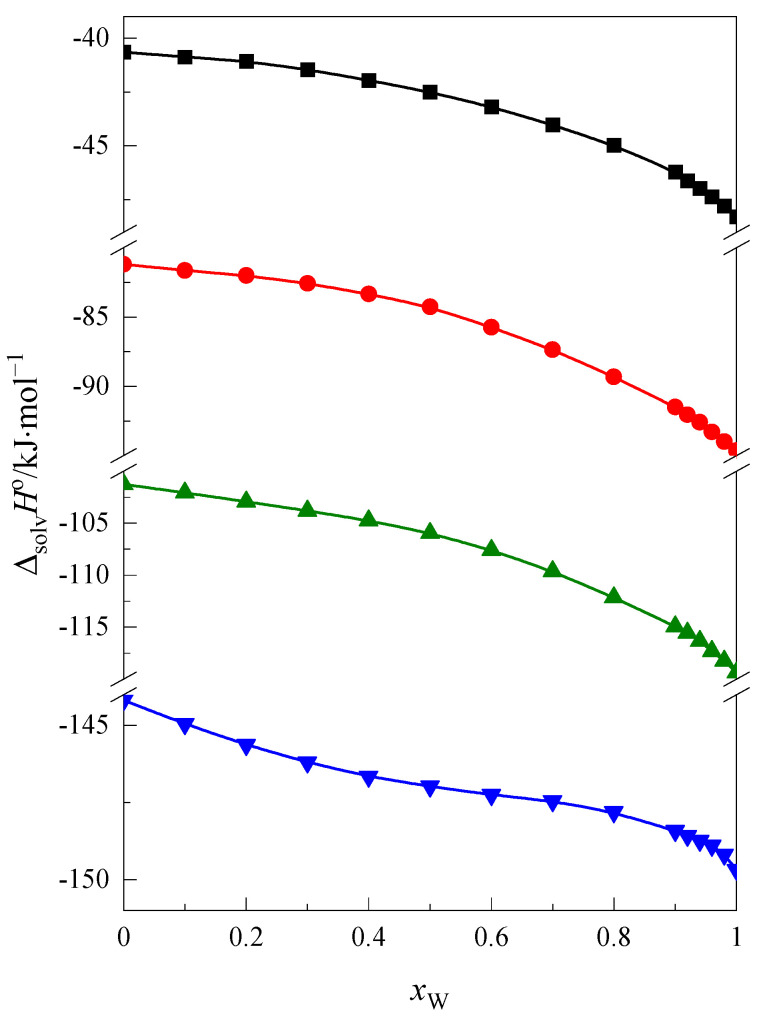
The standard solvation enthalpy of 1,4-dioxane, ■; 12C4 ●; 15C5, ▲; and 18C6, ▼ in the F + W mixture at 298.15 K.

In Figure 3 the standard enthalpy of solvation of cyclic ethers (1,4-dioxane [16] and crown ethers: 12C4 [16], 15C5 and 18C6) as a function of *x*_W_ $\Delta_{\mathrm{solv}}H^{o}=f(x_{W})$ is presented.

As is seen in Figure 3 the standard enthalpy of solvation is more negative the larger the ring of cyclic ethers is. The function $\Delta_{\mathrm{solv}}H^{o}=f(x_{W})$ decreases with an increase of the water content in the mixture. It is connected with the process of solvation and hydrophobic hydration of cyclic ethers. The range of changes in the values of this function are different and are presented in Table 1. 

The values of change in the enthalpy of solvation for 1,4-dioxane, 12C4 and 15C5 increase with increase of the ring size of cyclic ethers. In the case of 18C6, this change is very small. This clearly indicates differences in the interactions between the 1,4-dioxane, 12C4 and 15C5 molecules and between the 18C6 molecules and the mixed solvent molecules. As we wrote in previous part of this publication, it can be assumed that in addition to the effect of hydrophobic hydration, 18C6 forms complexes with water [38,39,40,41,42,43] and formamide molecules, hence such small change in this function.

### 2.2. The Preferential Solvation of Cyclic Ethers

In the case of the dissolution of molecules which exhibit hydrophobic character (cyclic ethers) in the mixtures of hydrophilic solvents (formamide) and water, the thermal effect of dissolution can consist of the two effects: the enthalpic effect of hydrophobic hydration and the enthalpic effect of preferential solvation. Therefore, the standard enthalpy of solution can be described by Equation (3). This equation is analogous to the equation underlying the preferential solvation model proposed by Covington et al. [44,45] and later modified by Balk and Somsen [46]. This model used for consideration in relation to cyclic ethers was discussed in detail in our previous publication [16].

(3) $$\Delta_{\mathrm{sol}}H^{o}(F+W)=x_{w}\Delta_{\mathrm{sol}}H^{o}\left(W\right)+x_{F}\Delta_{\mathrm{sol}}H^{o}\left(F\right)+(x_{w}^{n}-x_{w})Hb\left(W\right)+\Delta H^{*}(F+W)$$

where: 

$\Delta_{\mathrm{sol}}H^{o}(F+W)$, $\Delta_{\mathrm{sol}}H^{o}\left(W\right)$, $\Delta_{\mathrm{sol}}H^{o}\left(F\right)$—the standard dissolution enthalpy of hydrophobic substance in the mixed solvent, in water and formamide, respectively,

$x_{F}=(1-x_{w})$—the molar fraction of formamide in the mixed solvent,

*Hb*(W)—the enthalpic effect of hydrophobic hydration of the solute in pure water, 

$(x_{w}^{n}-x_{w})Hb\left(W\right)$—the factor related to the hydrophobic hydration process in the mixture, 

$\Delta H^{*}(F+W)$—the enthalpic effect of interactions in solution different from the hydrophobic hydration of the dissolution substance, 

*n*—does not have special meaning.

Using Equation (4) the value of the function: $\Delta H^{*}(F+W)$ can be calculated.

(4) $$\Delta H^{*}(F+W)=\Delta_{\mathrm{sol}}H^{o}\left(W\right)-\text{\{}x_{w}\Delta_{\mathrm{sol}}H^{o}\left(W\right)+x_{F}\Delta_{\mathrm{sol}}H^{o}(F)\text{\}}+(x_{w}^{n}-x_{w})Hb\left(W\right)$$

The details of the calculation of *Hb*(W) have been given in our previous paper [47]. Calculations of the function value were carried out using the values of parameters *n* and *Hb*(W) for 15C5 and 18C6 previously presented by us [17] in the mixture of water and *N*,*N*-dimethylformamide (DMF). For this the cage model of hydrophobic hydration proposed by Mastroianni, Pikal, and Lindenbaum [48] and the enthalpy of solution of 15C5 and 18C6 in the mixtures of formamide and water presented in this paper were used. DMF is a solvent almost neutral from the point of view of hydrophobic-hydrophilic character and in the mixture with water can be used as a solvent for the study of enthalpic effect of the hydrophobic hydration [47,48]. Using the cage model, the enthalpy of solution of hydrophobic substance (15C5 and 18C6) in the mixed solvent of DMF and water can be described by Equation (5).

(5) $$\Delta_{\mathrm{sol}}H^{o}(\mathrm{DMF}+W)=(1-x_{w})\Delta_{\mathrm{sol}}H^{o}\left(\mathrm{DMF}\right)+x_{w}\Delta_{\mathrm{sol}}H^{o}\left(W\right)+(x_{w}^{n}-x_{w})Hb\left(W\right)$$

where: Δ_sol_*H*^o^(DMF + W), Δ_sol_*H*^o^(DMF), Δ_sol_*H*^o^(W) are the enthalpy of solution of hydrophobic substance (15C5 or 18C6) in the DMF + W mixture, in DMF and water, respectively, *x*_w_ is the mol fraction of water, *Hb*(W) is the enthalpic effect of hydrophobic hydration of cyclic ethers in pure water, *n* does not have special meaning. 

**Figure 4:**
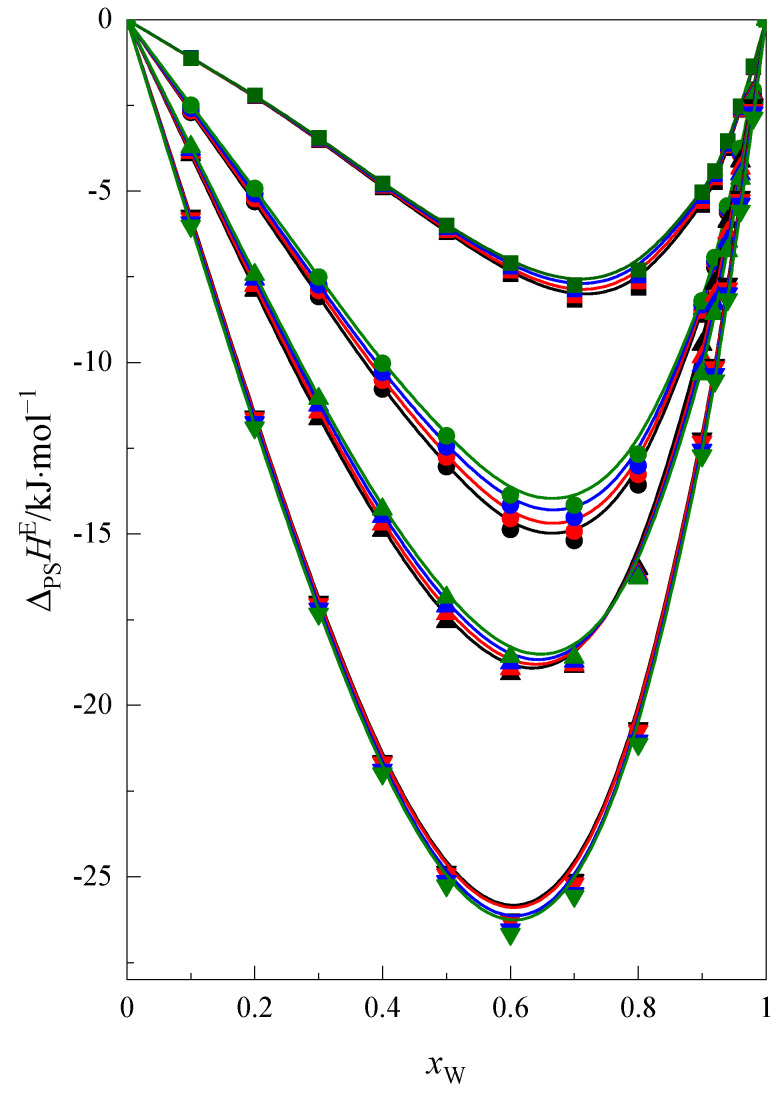
The enthalpic effect of the preferential solvation of cyclic ethers: 1,4-dioxane, ■ [16]; 12C4 ● [16]; 15C5, ▲; and 18C6, ▼ in the F + W mixture at: 293.15 K (black line), 298.15 K (red line), 303.15 K (blue line), 308.15 K (green line).

The function $\Delta H^{*}(F+W)=f(x_{w})$ calculated using Equation (4) for 15C5 and 18C6 is presented in Figure 4. For comparison in Figure 4 the analogous results for 1,4-dioxane [16] and 12-crown-4 [16] are also presented. As seen in Figure 4, the values of function $\Delta H^{*}(F+W)=f(x_{w})$ are negative within the whole mixed solvent composition range. It follows that the calculated enthalpic effect $\Delta H^{*}(F+W)$ is equivalent to the enthalpy effect of preferential solvation $\Delta_{\mathrm{PS}}H^{E}(F+W)$ [16,46] and the cyclic ethers molecules are preferentially solvated by the molecules of water or formamide. 

The exothermic enthalpic effect of preferential solvation $\Delta_{\mathrm{PS}}H^{E}(F+W)$ increases with increasing ring size of the cyclic ether molecules (Figure 4). In addition, as shown in Figure 4, this effect decreases with increasing temperature for all investigated cyclic ethers, which is related to the increase in thermal motion of molecules and the weakening of the interactions of dissolved molecules with molecules of the mixed solvent.

The analysis of the theory of preferential solvation used by us [44,45,49] can give an answer which water or formamide molecules preferentially solvate the cyclic ethers molecules. 

In this theory, analysis of the changes in the structure of the solute solvation sphere (S) caused by the change in the mixed solvent composition are presented. In the case when molecules of one of the components of the mixed solvent begin to dominate in the solvation sphere of the solute molecules (S), the changes can be described by a series of equilibria (Equation (6)) characterized by an equilibrium constant *K*_i_:

S(W*_i_*_−1_F*_r_*_+1−*i*_) + W ⇆ S(W*_i_*F*_r_*_−*i*_) + F  where (1 ≤ *i* ≤ *r*) (6)

It is assumed that the changes in Gibbs energy, which accompany the replacement of one F molecule with a W molecule, are the same for all the equilibria. The equilibrium constant of the *i* process may be expressed as follows:

(7) $$K_{i}=K^{1/r}[(r+1-i)/i]$$

where: *K*—the overall constant of equilibrium of all processes, $K=\overset{r}{\underset{i=1}{\Pi }}K_{i}$, *r* = $r_{W}$ + $r_{F}$, where $r_{w}$ and $r_{F}$ are the number of W and F molecules in the solute (S) solvation shell, respectively.

The enthalpic effect of the preferential solvation of the process presented by Equations (6) and (7) is described by Equation (8).

(8) $$\Delta_{\mathrm{PS}}H^{E}(F\;+\;W)=rRT\;\left[\left\{\frac{1-x_{W}}{\left(1-x_{W}\right)+K^{1/r}\cdot x_{W}}\right\}-\left(1-x_{W}\right)\right]\cdot \ln K^{1/r}$$

Using the Equation (8) and the method of non-linear regression the values of parameters *r* and *K*^1/*r*^ were calculated at four temperatures and they are presented in Table 2. 

As is seen in Table 2, the *r* parameter values are higher for 18C6 than those for 15C5. For both crown ethers, *r* values decrease with increasing temperature similarly to 1,4-dioxane and 12C4 [16]. 

As seen in Table 2, the values of the parameter *K*^1/*r*^ is lower than one within the investigated range of temperature, which means that the molecules of cyclic ether are preferentially solvated by the formamide molecules [46]. Probably the molecules of cyclic ethers interact in a specific way through hydrogen bonding with formamide molecules. Therefore, Equation (4) describing the preferential solvation process by water molecules is correct but in the opposite direction with the equilibrium constant *K*′ = 1/*K*. The calculated values of *K*′ are listed in Table 2. Considering the analogous data for 1,4-dioxane [16] and 12C4 [16], it can be seen that the equilibrium constants of the preferential solvation process increase with the increase of the ring size of the cyclic ethers and decrease with the increase of temperature. 

The thermodynamic function (Gibbs’ energy, enthalpy and entropy) of transfer molecules of cyclic ethers from W to the mixed solvent F + W in the preferential solvation process can be calculated using the analogous procedure as presented in the paper of Balk and Somsen [46] and Equations (9)–(11).

(9) $$\Delta_{\mathrm{tr}}G(W\to F\;+\;W)=-rRT\ln[K^{-1/r}x_{F}+x_{W}]$$

(10) $$\Delta_{\mathrm{tr}}H\left(W\to F\;+\;W\right)=rRT\left[\frac{1-x_{W}}{\left(1-x_{W}\right)+K^{1/r}x_{W}}\right]\cdot \ln K^{1/r}$$

(11) $$T\Delta_{\mathrm{tr}}S{\left(W\to \;F\;+\;W\right)}_{\left(A\right)}=-r_{W}RT\ln\left(\frac{r_{W}}{rx_{W}}\right)-r_{F}RT\ln\left(\frac{r_{F}}{r\left(1-x_{W}\right)}\right)$$

(12) $$T\Delta_{\mathrm{tr}}S{(W\to \;F\;+\;W)}_{\left(B\right)}=\Delta_{\mathrm{tr}}H(W\to \;F\;+\;W)-\Delta_{\mathrm{tr}}G(W\to \;F\;+\;W)$$

Using Equation (12) the values of $T\Delta_{\mathrm{tr}}S{\left(W\to \;F\;+\;W\right)}_{\left(B\right)}$ have been calculated. Then the values of the number of water molecules in the solvation sphere, *r*_W_, have been calculated using Equation (11) by choosing the *r*_W_ values so that equality was met: $T\Delta_{\mathrm{tr}}S{\left(W\to \;F\;+\;W\right)}_{\left(B\right)}$ = $T\Delta_{\mathrm{tr}}S{\left(W\to \;F\;+\;W\right)}_{\left(A\right)}$. Next, the values of the number of molecules of formamide in solvation sphere, *r*_F_, and the mole fraction of formamide, *y*_F_, in the solvation sphere of 1,4-dioxane, 12C4, 15C5 and 18C6 have been calculated. Tables S1–S16 with detailed calculations are in Supplementary Materials. As is seen in Tables S1–S16, the *y*_F_ values are temperature independent (within the error) for the individual cyclic ethers and are presented in Table 3 as a mean value of *y*_F_ at four temperatures. Additionally in Table 3 the mole fraction of water, *x*_W_, and formamide, *x*_F_, in the mixture W + F and in solvation sphere of cyclic ethers, *y*_F_, at *T* = 298.15 K is presented.

The uncertainty of the mole fraction *x*_W_ and *x*_F_ is equal to ±1 × 10^−3^. 

**Figure 5:**
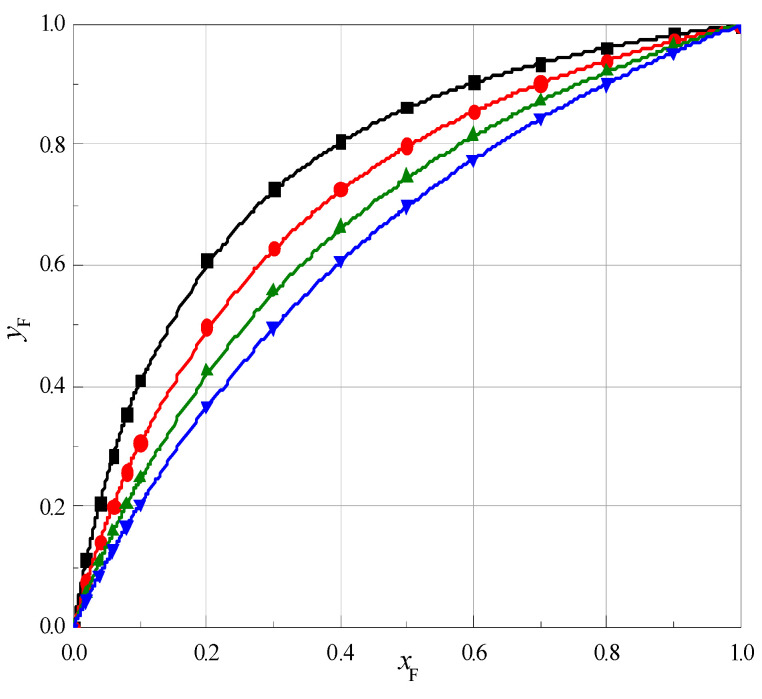
Dependence of the mole fraction of formamide in the solvation sphere ($y_{F}$) of cyclic ethers: ■, 1,4-dioxane; ●, 12C4; ▲, 15C5; ▼, 18C6, as a function of the mole fraction of formamide (*x*_F_) in the mixture F + W at 298.15 K.

A graphic illustration of the relationship $y_{F}=f\left(x_{F}\right)$ is shown in Figure 5. As seen in Table 3 and Figure 5 the mole fractions of formamide in the solvation sphere (*y*_F_) are higher than in the mole fraction of formamide in the mixture F + W (*x*_F_). Thus, it is clear that cyclic ethers are preferentially solvated by formamide molecules. Moreover, it can be observed that mole fraction of formamide in the solvation sphere decreases with the increasing cyclic ethers ring. 

In the case of 18C6 this is understandable as it most likely forms complexes with formamide in the solution. Probably the reason for this is the formation of complexes of 18C6 molecules with water [38,39,40,41,42,43] and formamide, which we wrote about in the earlier part of this work. In the case of 12C4 and 15C5, similar but weaker interactions of this type cannot be ruled out. Unfortunately, we have not found such reports in the literature.

In the systems previously investigated by us (glymes + DMF + methanol [26], glymes + DMF + propan-1-ol [26] and cyclic ethers + DMF + propan-1-ol [27]) has been observed that glymes molecules are preferentially solvated by DMF molecules in the DMF + propan-1-ol mixture [26], and by methanol molecules in the DMF + methanol mixture [26], as well as, the cyclic ethers molecules are preferentially solvated by DMF molecules in the DMF + propan-1-ol mixture. In the studied systems the dependence of mole fraction of the preferentially solvating solvent on the size of the solvated molecule was not observed. The mole fraction of the solvating solvent is constant (within error). We assume that the molecules of glymes and cyclic ethers do not form complexes with molecules of DMF, methanol and propan-1-ol. The shapes of the dissolution enthalpy curves and the lack of literature reports do not indicate such interactions. 

Based on the analysis, it can be concluded that the model we used, proposed by Covington and co-workers and modified by Balk and Somsen [44,45,46], can be successfully applied to the quantitative analysis of systems in which dissolved molecules do not form complexes with solvent molecules. However, in the case when the solute molecules form complexes with the solvent molecules and are additionally preferentially solvated by the mixed solvent molecules, the model can be used but only for qualitative analysis.

## 3. Experimental Section

### 3.1. Materials 

Suppliers, purity, a method of purification and water content in the compounds used for the measurements (15-crown-5, 18-crown-6 and formamide) and validation of calorimeter (urea potassium chloride) are shown in Table 4. To prepare the aqueous solutions double distilled water was used. 

### 3.2. Methods

The heat of solution of 15C5 and 18C6 in the mixture of formamide with water (F + W) was measured within the whole mole fraction range of the mixed solvent at [(293.15, 298.15, 303.15, 308.15) ± 0.01] K using an “isoperibol” type calorimeter as described in the literature [50]. The calorimeter was verified on the basis of the standard enthalpy of solution of urea and potassium chloride (KCl) (Calorimetric standard US, NBS) in water at 298.15 K [51,52]. The solution enthalpy of urea in water obtained by us from seven independent measurements was (15.31 ± 0.06) kJ mol^−1^ (literature data 15.31∙kJ mol^−1^ [53], 15.28 kJ mol^−1^ [54] and that for KCl in water was (17.55 ± 0.05) kJ mol^−1^ (literature data 17.58 kJ mol^−1^ [51,52]. 

For each investigation system six to eight independent measurements were performed. The uncertainties in the measured enthalpies did not exceed ±0.5% of the measured value. Since no concentration dependence was observed within the investigated concentration range, the enthalpy of dissolution of crown ethers, $\Delta_{\mathrm{sol}}H_{}^{o}$, was calculated as the average of six to eight measurements (Table 5 and Table 6).

## 4. Conclusions

The study of the enthalpy of solution of cyclic ethers (15C5 and 18C6) in the mixture of formamide and water (F + W) at four temperatures as well as their comparison with analogous data concerning 1,4-dioxane and 12C4 led to the following conclusion about the solvation of cyclic ethers.

1. The exothermic enthalpic effect of the solvation process increases with the increasing of size of the cyclic ethers ring.
2. Molecules of 1,4-dioxane, 12C4 15C5 and 18C6 are preferentially solvated by the molecules of formamide in the mixture of water and formamide.
3. The exothermic enthalpic effect of preferential solvation process decreases with increasing temperature for the cyclic ethers (1,4-dioxane, 12C4, 15C5 and 18C6).
4. The total number of W and F molecules in the solvation sphere of the molecules of cyclic ethers increases with increasing of ring size of ethers and does not depend on temperature.
5. The mole fraction of formamide present in the solvation shell of cyclic ethers is higher than that in the F + W mixture.
6. The 18C6 molecules most probably form complexes with formamide molecules in the solution of the F + W mixture. The same is true for 12C4 and 15C5, but to a lesser extent.
7. The model of preferential solvation, proposed by Covington et al. and modified by Balk and Somsen can be successfully applied to quantitative analysis of systems in which dissolved molecules do not form complexes with solvent molecules. In the case when the solute molecules form complexes with the solvent molecules and are additionally preferentially solvated by the mixed solvent molecules, this model can be used but only for qualitative analysis.

## Figures and Tables

**Table 1:** Changes in $\Delta_{\mathrm{solv}}H^{o}=f(x_{W})$ for cyclic ethers in the F + W mixture at 298.15 K.

	$\Delta_{\mathrm{solv}}H^{o}=f(x_{W})$/kJ·mol^−1^		
Cyclic Ethers	$\Delta_{\mathrm{solv}}H^{o}$ at *x*_W_ = 0	$\Delta_{\mathrm{solv}}H^{o}$ at *x*_W_ = 1	$\Delta_{\mathrm{solv}}H^{o}(x_{W}=0)-\Delta_{\mathrm{solv}}H^{o}(x_{W}=1)$
1,4-dioxane	−40.65	−48.28	7.63
12C4	−81.20	−94.63	13.43
15C5	−101.26	−119.37	18.11
18C6	−144.18	−149.68	5.50

**Table 2:** Parameters of Equation (8) for the preferential solvation of cyclic ethers in the F + W mixtures.

Cyclic Ether	*T/*K	*r*	*K* ^1/*r*^	*K*	*K*′ = 1/*K*	*R* ^2^
15C5	293.15	30.3 ± 0.4	0.36 ± 0.01	3.49 × 10^–14^	2.9 × 10^13^	0.9921
	298.15	26.0 ± 0.3	0.34 ± 0.01	6.44 × 10^–13^	1.6 × 10^12^	0.9936
	303.15	22.9 ± 0.3	0.32 ± 0.01	4.50 × 10^–12^	2.2 × 10^11^	0.9944
	308.15	20.3 ± 0.2	0.30 ± 0.01	2.46 × 10^–12^	4.1 × 10^10^	0.9952
18C6	293.15	61.3 ± 0.7	0.44 ± 0.01	1.38 × 10^–22^	7.2 × 10^21^	0.9949
	298.15	59.5 ± 0.6	0.43 ± 0.01	1.51 × 10^–22^	6.6 × 10^21^	0.9954
	303.15	57.6 ± 0.6	0.43 ± 0.01	7.79 × 10^–22^	1.3 × 10^21^	0.9958
	308.15	55.9 ± 0.5	0.42 ± 0.01	8.55 × 10^–22^	1.2 × 10^21^	0.9967

± is the standard deviation. *R*^2^ is the regression coefficient.

**Table 3:** The mole fraction of formamide (*y*_F_) in the solvation sphere of 1,4-dioxane, 12C4, 15C5 and 18C6, in the process of preferential solvation in dependency of the mole fraction of water (*x*_W_) or formamide (*x*_F_) in the mixture F + W.

		1,4-Dioxane	12C4	15C5	18C6
$x_{W}$ ^a^	$x_{F}$ ^b^	$y_{F}$ ^c^	$y_{F}$ ^c^	$y_{F}$ ^c^	$y_{F}$ ^c^
0.000	1.000	1.000	1.000	1.000	1.000
0.100	0.900	0.983 ± 0.000	0.973 ± 0.001	0.965 ± 0.003	0.954 ± 0.001
0.200	0.800	0.962 ± 0.001	0.942 ± 0.001	0.924 ± 0.005	0.903 ± 0.002
0.300	0.700	0.937 ± 0.001	0.904 ± 0.002	0.876 ± 0.008	0.844 ± 0.003
0.400	0.600	0.905 ± 0.002	0.858 ± 0.002	0.820 ± 0.012	0.777 ± 0.003
0.500	0.500	0.864 ± 0.003	0.801 ± 0.003	0.752 ± 0.015	0.699 ± 0.004
0.600	0.400	0.809 ± 0.004	0.729 ± 0.004	0.669 ± 0.017	0.608 ± 0.005
0.700	0.300	0.732 ± 0.005	0.634 ± 0.005	0.566 ± 0.019	0.499 ± 0.005
0.800	0.200	0.614 ± 0.006	0.502 ± 0.005	0.432 ± 0.019	0.368 ± 0.004
0.900	0.100	0.414 ± 0.007	0.310 ± 0.004	0.253 ± 0.015	0.205 ± 0.003
0.920	0.080	0.356 ± 0.006	0.260 ± 0.004	0.209 ± 0.013	0.168 ± 0.003
0.940	0.060	0.289 ± 0.006	0.205 ± 0.003	0.163 ± 0.011	0.129 ± 0.002
0.960	0.040	0.210 ± 0.005	0.144 ± 0.002	0.113 ± 0.008	0.088 ± 0.002
0.980	0.020	0.115 ± 0.003	0.076 ± 0.001	0.059 ± 0.004	0.045 ± 0.001
1.000	0.000	0.000	0.000	0.000	0.000

^a^ is the mole fraction of water in the F + W mixture. ^b^ is the mole fraction of formamide in the F + W mixture. ^c^ is the mole fraction of formamide in the solvation sphere of cyclic ethers in the process of preferential solvation in the F + W mixture.

**Table 4:** Materials.

Chemical Name	Source	Mole Fraction Purity ^a^	Purification Method	Mass Fraction of Water ^b^
Urea (U)	Sigma-Aldrich, (Poznan, Poland)	>0.995	recrystallization from ethanol and dried under reduced pressure to constant mass	−
Potassium chloride (KCl)	Sigma-Aldrich, (Poznan, Poland)	>0.99	drying under reduced pressure to constant mass	−
15-crown-5 (15C5)	Sigma-Aldrich, (Poznan, Poland)	0.98	drying under reduced pressure	1 × 10^−3^
18-crown-6 (18C6)	Sigma-Aldrich, (Poznan, Poland)	≥0.99	recrystallization from hexane and dried under reduced pressure	−
Formamide (F)	Sigma-Aldrich, (Poznan, Poland	>0.99	drying using 4A molecular sieves and calcium oxide and distillation under reduced pressure	3 × 10^−4^

^a^ Declared by the supplier. ^b^ Determined by the Karl Fischer method.

**Table 5:** Standard molar enthalpy of solution ($\Delta_{\mathrm{sol}}H_{}^{o}$) and molality (*m*) of 15C5 in the F + W mixtures at *T* = (293.15, 298.15, 303.15, 308.15) K.

	*T* = 293.15 K		*T* = 298.15 K		*T* = 303.15 K		*T* = 308.15 K	
$x_{w}$ ^a^	$\frac{m^{b}\cdot 10_{}^{3}}{\mathrm{mol}\cdot {\mathrm{kg}}^{-1}}$	$\frac{\Delta_{\mathrm{sol}}H_{}^{o}}{\mathrm{kJ}\cdot {\mathrm{mol}}^{-1}}$	$\frac{m^{b}\cdot 10_{}^{3}}{\mathrm{mol}\cdot {\mathrm{kg}}^{-1}}$	$\frac{\Delta_{\mathrm{sol}}H_{}^{o}}{\mathrm{kJ}\cdot {\mathrm{mol}}^{-1}}$	$\frac{m^{b}\cdot 10_{}^{3}}{\mathrm{mol}\cdot {\mathrm{kg}}^{-1}}$	$\frac{\Delta_{\mathrm{sol}}H_{}^{o}}{\mathrm{kJ}\cdot {\mathrm{mol}}^{-1}}$	$\frac{m^{b}\cdot 10_{}^{3}}{\mathrm{mol}\cdot {\mathrm{kg}}^{-1}}$	$\frac{\Delta_{\mathrm{sol}}H_{}^{o}}{\mathrm{kJ}\cdot {\mathrm{mol}}^{-1}}$
0.000	2.42–2.60	−22.00 ± 0.04 ^c^	2.31–2.34	−21.69 ± 0.04	2.62–2.88	−21.40 ± 0.06	3.62–4.62	−21.00 ± 0.05
0.100	3.07–3.30	−22.85 ± 0.04	2.24–2.35	−22.50 ± 0.05	1.99–2.90	−22.15 ± 0.06	3.80–5.36	−21.71 ± 0.05
0.200	2.84–3.15	−23.75 ± 0.08	2.22–2.26	−23.36 ± 0.04	2.61–3.18	−22.95 ± 0.04	2.87–6.70	−22.49 ± 0.08
0.300	2.58–2.99	−24.67 ± 0.05	2.26–2.29	−24.24 ± 0.04	2.42–3.25	−23.83 ± 0.04	1.57–1.62	−23.34 ± 0.04
0.400	2.40–3.20	−25.60 ± 0.06	2.17–2.21	−25.18 ± 0.06	2.55–3.26	−24.76 ± 0.08	1.46–1.50	−24.25 ± 0.04
0.500	2.58–2.71	−26.86 ± 0.06	2.13–2.16	−26.37 ± 0.06	2.72–2.74	−25.90 ± 0.06	1.05–1.63	−25.34 ± 0.06
0.600	3.03–3.12	−28.48 ± 0.08	2.23–2.28	−28.01 ± 0.04	2.73–3.10	−27.53 ± 0.06	1.56–2.12	−26.95 ± 0.06
0.700	2.60 –2.66	−30.58 ± 0.04	2.91–3.02	−30.06 ± 0.06	2.76–3.42	−29.52 ± 0.09	1.82–1.85	−28.87 ± 0.06
0.800	2.52–3.58	−33.10 ± 0.04	1.82–1.97	−32.57 ± 0.04	2.88–3.06	−31.97 ± 0.05	1.53–1.91	−31.30 ± 0.06
0.900	3.88 –3.89	−36.00 ± 0.04	2.61–2.79	−35.37 ± 0.04	2.67–2.69	−34.64 ± 0.09	1.38–1.58	−33.90 ± 0.09
0.920	3.16–3.30	−36.70 ± 0.04	2.27–2.35	−35.98 ± 0.06	1.53–1.97	−35.18 ± 0.04	1.64–1.82	−34.40 ± 0.08
0.940	3.47–3.59	−37.60 ± 0.06	2.26–2.58	−36.78 ± 0.05	1.69–1.75	−35.88 ± 0.06	1.60–2.09	−35.03 ± 0.08
0.960	3.38–3.43	−38.77 ± 0.05	2.94–3.07	−37.74 ± 0.05	1.32–1.66	−36.67 ± 0.05	2.14–3.81	−35.60 ± 0.06
0.980	3.18–3.81	−39.98 ± 0.04	2.14–2.23	−38.68 ± 0.04	1.20–1.97	−37.40 ± 0.06	3.39–3.79	−36.17 ± 0.04
1.000	1.16–2.48	−41.24 ± 0.04	2.15–2.32	−39.85 ± 0.02	1.58–3.52	−38.37 ± 0.03	1.37–1.52	−37.03 ± 0.04
				−40.64 [47]				
				−39.71 [33]				

^a^ $x_{w}$ is the mole fraction of water in solvent mixture. ^b^ *m* is the concentration range investigated of 15C5 obtained from six to eight independent measurements. ^c^ ± is the uncertainty. The uncertainty of the mole fraction *x*_W_ is equal to ±1 × 10^−3^. The uncertainty of molality is equal to ±1.5 × 10^−5^ mol·kg^−1^.

**Table 6:** Standard molar enthalpy of solution ($\Delta_{\mathrm{sol}}H_{}^{o}$) and molality (*m*) of 18C6 in the F + W mixtures at *T* = (293.15, 298.15, 303.15, 308.15) K.

	*T* = 293.15 K		*T* = 298.15 K		*T* = 303.15 K		*T* = 308.15 K	
$x_{w}$ ^a^	$\frac{m^{b}\cdot 10_{}^{3}}{\mathrm{mol}\cdot {\mathrm{kg}}^{-1}}$	$\frac{\Delta_{\mathrm{sol}}H_{}^{o}}{\mathrm{kJ}\cdot {\mathrm{mol}}^{-1}}$	$\frac{m^{b}\cdot 10_{}^{3}}{\mathrm{mol}\cdot {\mathrm{kg}}^{-1}}$	$\frac{\Delta_{\mathrm{sol}}H_{}^{o}}{\mathrm{kJ}\cdot {\mathrm{mol}}^{-1}}$	$\frac{m^{b}\cdot 10_{}^{3}}{\mathrm{mol}\cdot {\mathrm{kg}}^{-1}}$	$\frac{\Delta_{\mathrm{sol}}H_{}^{o}}{\mathrm{kJ}\cdot {\mathrm{mol}}^{-1}}$	$\frac{m^{b}\cdot 10_{}^{3}}{\mathrm{mol}\cdot {\mathrm{kg}}^{-1}}$	$\frac{\Delta_{\mathrm{sol}}H_{}^{o}}{\mathrm{kJ}\cdot {\mathrm{mol}}^{-1}}$
0.00	1.02–1.08	−17.35 ± 0.04 ^c^	1.09–1.69	−16.43 ± 0.06	1.23–1.43	−15.53 ± 0.05	1.13–1.71	−14.55 ± 0.06
0.10	1.23–1.74	−17.65 ± 0.04	1.31–1.69	−16.75 ± 0.09	1.58–2.24	−15.87 ± 0.06	1.14–1.27	−14.92 ± 0.06
0.20	1.07–1.73	−18.19 ± 0.08	1.03–2.01	−17.22 ± 0.04	1.94–1.53	−16.26 ± 0.05	1.00–1.43	−15.29 ± 0.04
0.30	1.17–1.47	−18.66 ± 0.06	2.04–2.26	−17.67 ± 0.09	0.92–1.47	−16.66 ± 0.06	1.03–1.53	−15.63 ± 0.06
0.40	1.21–1.48	−19.15 ± 0.05	1.72–2.73	−18.12 ± 0.06	1.22–1.51	−17.09 ± 0.04	1.01–1.58	−16.00 ± 0.08
0.50	1.12–1.15	−19.48 ± 0.04	1.41–1.73	−18.46 ± 0.05	1.72–1.90	−17.35 ± 0.08	0.78–1.48	−16.25 ± 0.06
0.60	0.87–1.74	−19.84 ± 0.06	1.40–1.88	−18.77 ± 0.06	2.26–2.71	−17.63 ± 0.05	1.52–2.51	−16.50 ± 0.04
0.70	0.97–1.31	−20.23 ± 0.06	1.70–2.30	−19.15 ± 0.05	1.95–2.49	−17.95 ± 0.05	1.42–2.11	−16.70 ± 0.06
0.80	1.52–1.60	−20.72 ± 0.04	1.68–2.18	−19.46 ± 0.06	2.06–2.54	−18.15 ± 0.04	1.08–1.40	−16.78 ± 0.04
0.90	1.37–1.58	−21.45 ± 0.04	1.80–2.68	−19.94 ± 0.06	2.84–3.23	−18.40 ± 0.05	1.17–1.31	−16.84 ± 0.06
0.92	1.04–1.45	−21.74 ± 0.05	2.00–2.67	−20.12 ± 0.06	3.97–4.91	−18.51 ± 0.06	0.98–1.81	−16.90 ± 0.08
0.94	1.20–1.30	−22.02 ± 0.08	2.53–3.16	−20.39 ± 0.04	2.06–3.11	−18.68 ± 0.04	1.54–1.92	−16.97 ± 0.06
0.96	1.41–2.00	−22.37 ± 0.05	1.43–1.82	−20.60 ± 0.05	2.10–3.02	−18.80 ± 0.08	1.44–1.92	−17.05 ± 0.04
0.98	0.96–1.08	−22.80 ± 0.04	1.34–1.80	−20.99 ± 0.06	2.16–3.39	−19.10 ± 0.06	1.41–1.77	−17.20 ± 0.06
1.00	5.43–6.52	−23.65 ± 0.02	1.92–2.44	−21.55 ± 0.03	2.55–5.87	−19.57 ± 0.02	2.48–4.64	−17.42 ± 0.02
				−21.58 [17]				
				−21.54 [33]				
				−21.36 [55]				

^a^ $x_{w}$ is the mole fraction of water in solvent mixture. ^b^ *m* is the concentration range investigated of 15C5 obtained from six to eight independent measurements. ^c^ ± is the uncertainty. The uncertainty of the mole fraction *x*_W_ is equal to ±1 × 10^−3^. The uncertainty of molality is equal to ±1.5 × 10^−5^ mol·kg^−1^

## Data Availability

Not applicable.

## Supplementary Materials

The following supporting information can be downloaded at: https://www.mdpi.com/article/10.3390/molecules28052169/s1, Table S1–S4: Total number of water (W) and formamide (F) molecules in the solvation sphere of 1,4-dioxane r = (rW + rF), mole fraction of formamide (F) in the solvation sphere of the solute (yF), entropic factor of the transfer entropy of 1,4-dioxane in the process of preferential solvation at T = 293.15 K, 298.15 K, 303.15 K, 308.15 K in dependency of the mole fraction of water (xW) or formamide (xF) in the mixture F + W; Table S5–S8: Total number of water (W) and formamide (F) molecules in the solvation sphere of 12C4 r = (rW + rF), mole fraction of formamide (F) in the solvation sphere of the solute (yF), entropic factor of the transfer entropy of 12C4 in the process of preferential solvation at T = 293.15 K, 298.15 K, 303.15 K, 308.15 K in dependency of the mole fraction of water (xW) or formamide (xF) in the mixture F + W; Table S9–S12: Total number of water (W) and formamide (F) molecules in the solvation sphere of 15C5 *r* = (*r*_W_ + *r*_F_), mole fraction of formamide (F) in the solvation sphere of the solute (*y*_F_), entropic factor of the transfer entropy of 15C5 in the process of preferential solvation at T = 293.15 K, 298.15 K, 303.15 K, 308.15 K in dependency of the mole fraction of water (*x*_W_) or formamide (*x*_F_) in the mixture F + W. Table S13–S16: Total number of water (W) and formamide (F) molecules in the solvation sphere of 18C6 r = (rW + rF), mole fraction of formamide (F) in the solvation sphere of the solute (yF), entropic factor of the transfer entropy of 18C6 in the process of preferential solvation at T = 293.15 K, 298.15 K, 303.15 K, 308.15 K in dependency of the mole fraction of water (xW) or formamide (xF) in the mixture F + W.

## References

[1] Parajó J.J., Otero-Mato J.M., Lobo Ferreira A.I.M.C., Varela L.M., Santos L.M.N.B.F. (2022). Enthalpy of solvation of alkali metal salts in a protic ionic liquid: Effect of cation charge and size. J. Mol. Liq..

[2] Rakipov I.T., Semenov K.N., Petrov A.A., Akhmadiyarov A.A., Khachatrian A.A., Fakhurtdinova A.R., Solomonov B.N. (2021). Thermochemistry of solution, solvation and hydrogen bonding of linear and cyclic ethers in solvents. Thermochim. Acta.

[3] Kuz’Mina I.A., Kovanova M.A., Udalova A.S. (2022). Solvation of dibenzo-18-crown-6 ether in water-aprotic solvents. J. Mol. Liq..

[4] Usacheva T.R., Volynkin V.A., Panyushkin V.T., Lindt D.A., Pham T.L., Nguyen T.T.H., Le T.M.H., Alister D.A., Kabirov D.N., Kuranova N.N. (2021). Complexation of Cyclodextrins with Benzoic Acid in Water-Organic Solvents: A Solvation-Thermodynamic Approach. Molecules.

[5] Kamiyama T., Liu H.L., Kimura T. (2009). Preferential solvation of lysozyme by dimethyl sulfoxide in binary solutions of water and dimethyl sulfoxide. J. Therm. Anal. Calorim..

[6] Haghbakhsh R., Duarte A.R.C., Raeissi S. (2021). Viscosity Investigations on the Binary Systems of (1 ChCl:2 Ethylene Glycol) DES and Methanol or Ethanol. Molecules.

[7] Rakipov I.T., Petrov A.A., Akhmadiyarov A.A., Khachatrian A.A., Mukhametzyanov T.A., Solomonov B.N. (2021). Thermochemistry of Solution, Solvation, and Hydrogen Bonding of Cyclic Amides in Proton Acceptor and Donor Solvents. Amide Cycle Size Effect. Molecules.

[8] Marcus Y. (2008). On the preferential solvation of drugs and PAHs in binary solvent mixtures. J. Mol. Liq..

[9] Kalidas C., Raghunath R. (1995). Preferential solvation of silver(I)-cryptand-2,2,2 perchlorate complex in water + acetonitrile and methanol + acetonitrile mixtures. J. Electroanal. Chem..

[10] Marcus Y. (2007). Preferential solvation of ions in mixed solvents. 6: Univalent anions in aqueous organic solvents according to the inverse Kirkwood-Buff integral (IKBI) approach. J. Chem. Thermodyn..

[11] Ben-Naim A. (1990). Preferential solvation in two- and in three-component systems. Pure Appl. Chem..

[12] Sun H., Du C. (2023). Dissolution of 5-azacytidine in aqueous solution of alcohols at various temperatures: Preferential solvation and thermodynamic analysis. J. Chem. Thermodyn..

[13] Guo Q., Shi W., Zhao H., Li W., Han G., Farajtabar A. (2023). Solubility, solvent effect, preferential solvation and DFT computations of 5-nitrosalicylic acid in several aqueous blends. J. Chem. Thermodyn..

[14] Zhang C., Liu Y., Song F., Wang J. (2022). Inter-/intra-molecular interactions, preferential solvation, and dissolution and transfer property for tirofiban in aqueous co-solvent mixtures. J. Mol. Liq..

[15] Cong Y., Du C., Xing K., Bian Y., Li X., Wang M. (2022). Research on dissolution of actarit in aqueous mixtures: Solubility determination and correlation, preferential solvation, solvent effect and thermodynamics. J. Mol. Liq..

[16] Jóźwiak M., Kosiorowska M.A. (2010). Effect of Temperature on the Process of Hydrophobic Hydration. Part I. Hydrophobic Hydration of 1,4-Dioxane and 12-Crown-4 Ethers. J. Chem. Eng. Data.

[17] Jóźwiak M., Kosiorowska M.A., Wasiak M. (2010). Effect of Temperature on the Process of Hydrophobic Hydration. Part II. Hydrophobic Hydration of 15-Crown-5 and 18-Crown-6 Ethers. J. Chem. Eng. Data.

[18] Jóźwiak M. (2004). The effect of properties of water-organic solvent mixtures on the solvation enthalpy of 12-crown-4, 15-crown-5, 18-crown-6 and benzo-15-crown-55 ethers at 298.15 K. Thermochim. Acta.

[19] Nayak J.N., Aralaguppi M.I., Naidu B.V.K., Aminabhavi T.M. (2004). Thermodynamic Properties of Water + Tetrahydrofuran and Water + 1,4-Dioxane Mixtures at (303.15, 313.15, and 323.15) K. J. Chem. Eng. Data.

[20] Junk P.C. (2008). Crown ethers as stabilising ligands for oxonium ions. New J. Chem..

[21] Hilderbrand A.E., Myung S., Clemmer D.E. (2006). Exploring Crown Ethers as Shift Reagents for Ion Mobility Spectrometry. Anal. Chem..

[22] Sisson A.L., Shah M.R., Bhosale S., Matile S. (2006). Synthetic ion channels and pores (2004–2005). Chem. Soc. Rev..

[23] Suzumura A., Paul D., Sugimoto H., Shinoda S., Julian R.R., Beauchamp J.L., Teraoka J., Tsukube H. (2005). Cytochrome c-Crown Ether Complexes as Supramolecular Catalysts: Cold-Active Synzymes for Asymmetric Sulfoxide Oxidation in Methanol. Inorg. Chem..

[24] Jamali S.H., Ramdin M., Becker T.M., Rinwa S.K., Buijs W., Vlugt T.J.H. (2017). Thermodynamic and Transport Properties of Crown-Ethers: Force Field Development and Molecular Simulations. J. Phys. Chem. B.

[25] Ullah F., Khan T.A., Iltaf J., Anwar S., Khan M.F.A., Khan M.R., Ullah S., Rehman M.F.U., Mustaqeem M., Kotwica-Mojzych K. (2022). Heterocyclic Crown Ethers with Potential Biological and Pharmacological Properties: From Synthesis to Applications. Appl. Sci..

[26] Jóźwiak M., Łudzik K., Cokot M., Jóźwiak A., Kłys A. (2020). Solvation enthalpy of selected glymes in the mixtures of N,N-dimethylformamide with propan-1-ol or methanol at 298.15 K. The solvent contribution to the solvation enthalpy of glymes. J. Mol. Liq..

[27] Jóźwiak M., Urban A., Tyczyńska M. (2021). Effect of properties of N,N-dimethylformamide + propan-1-ol mixtures on the solution enthalpy of selected cyclic ethers in these mixtures at 298.15 K. The contribution of solvent to the enthalpy of solvation of cyclic ethers. J. Mol. Liq..

[28] Pullman A., Berthod H., Giessner-Prettre C., Hinton J.F., Harpool D. (1978). Hydrogen bonding in pure and aqueous formamide. J. Am. Chem. Soc..

[29] Riddick J.A., Bunger W.B., Sakano T.K. (1986). Organic Solvents.

[30] Shedlovskiy D., Shcherbik N., Pestov D.G. (2017). One-step hot formamide extraction of RNA from Saccharomyces cerevisiae. RNA Biol..

[31] Höhn A., Staff U. (2014). Formamide. Kirk-Othmer encyclopedia of chemical technology.

[32] Brocos P., Calvo E., Bravo R., Pintos M., Amigo A., Roux A.H., Roux-Desgranges G. (1999). Heat Capacities, Excess Enthalpies, and Volumes of Mixtures Containing Cyclic Ethers. 3. Binary Systems {Tetrahydrofuran, Tetrahydropyran, 1,4-Dioxane, or 1,3-Dioxolane + Cyclohexane or Toluene}. J. Chem. Eng. Data.

[33] Briggner L.-E., Wadsö I. (1990). Some thermodynamic properties of crown ethers in aqueous solution. J. Chem. Thermodyn..

[34] Cabani S., Gianni P., Mollica V., Lepori L. (1981). Group Contributions to the Thermodynamic Properties of Non-Ionic Organic Solutes in Dilute Aqueous Solution. J. Solut. Chem..

[35] Vögtle F., Müller W.M., Weber E. (1980). Komplexe zwischen Neutralmolekülen, VII. Kronenether als Wirtssubstanzen für organische Gastmoleküle. Chem. Ber..

[36] Watson W.H., Galloy J., Grossie D.A., Voegtle F., Mueller W.M. (1984). Host-guest complex chemistry. Structures of 18-crown-6 and diaza-18-crown-6 with neutral molecules. J. Org. Chem..

[37] Mosier-Boss P.A., Popov A.I. (1985). NMR and infrared studies of the complexation reaction of 18-crown-6 with some organic solvents. J. Am. Chem. Soc..

[38] Matsuda H., Yamada Y., Kanamori K., Kudo Y., Takeda Y. (1991). On the Facilitation Effect of Neutral Macrocyclic Ligands on the Ion Transfer across the Interface between Aqueous and Organic Solutions. I. Theoretical Equation of Ion-Transfer-Polarographic Current-Potential Curves and Its Experimental Verification. Bull. Chem. Soc. Jpn..

[39] Kowall T., Geiger A. (1994). Molecular Dynamics Simulation Study of 18-Crown-6 in Aqueous Solution. 1. Structure and Dynamics of the Hydration Shell. J. Phys. Chem..

[40] Patil K.J., Pawar R.B., Patil P.D. (2000). The studies of viscosity behaviour in aqueous 18-crown-6 solutions at 25 °C. J. Mol. Liq..

[41] Dagade D., Pawar R., Patil K. (2004). Viscosity Behavior of 18-Crown-6 in Aqueous and Carbon Tetrachloride Solutions at Different Temperatures and at Ambient Pressure. J. Chem. Eng. Data.

[42] Bryan S.A., Willis R.R., Moyer B.A. (1990). Hydration of 18-crown-6 in carbon tetrachloride: Infrared spectral evidence for an equilibrium between monodentate and bidentate forms of bound water in the 1:1 crown-water adduct. J. Phys. Chem..

[43] Fukuhara K., Tachikake M., Matsumoto S., Matsuura H. (1995). Raman Spectroscopic Study of the Hydrates of 18-Crown-6. J. Phys. Chem..

[44] Covington A.K., Thain J.M. (1974). Nuclear magnetic resonance studies of preferential solvation. Part 4.—Thermodynamic treatment involving non-statistical distribution of solvated species. J. Chem. Soc. Faraday Trans. 1.

[45] Remerie K., Engberts J.B.F.N. (1983). Preferential solvation of nonelectrolytes in mixed aqueous solvents. A quantitative approach for.beta.-disulfones in 1,4-dioxane-water, 1,3-dioxane-water and dimethoxyethane-water in terms of the Covington theory. J. Phys. Chem..

[46] Balk R.W., Somsen G. (1985). Preferential solvation and hydrophobic hydration of polyols in mixtures of water and N,N-dimethylformamide. J. Phys. Chem..

[47] Jóźwiak M., Piekarski H. (1999). Heat of solution of 15-crown-5 ether in the mixtures of water with DMSO, DMF, DMA and HMPA at 298.15K. J. Mol. Liq..

[48] Mastroianni M.J., Pikal M.J., Lindenbaum S. (1972). Effect of dimethyl sulfoxide, urea, guanidine hydrochloride, and sodium chloride on hydrophobic interactions. Heats of dilution of tetrabutylammonium bromide and lithium bromide in mixed aqueous solvent systems. J. Phys. Chem..

[49] Covington A.K., Newman K.E. (1976). Thermodynamics of Preferential Solvation of Electrolytes in Binary Solvent Mixtures. Adv. Chem. Ser..

[50] Piekarski H., Waliszewski D. (1995). Hydration effect on urea-non-electrolyte enthalpic pair interaction coefficients. Dissolution enthalpies of urea in aqueous solution of alkoxyethanols at 298.15 K. Thermochim. Acta.

[51] Sabbah R., Xu A., Chickos J.S., Planas Leitão M.L., Roux M.V., Torres L.A. (1999). Reference materials for calorimetry and differential thermal analysis. Thermochim. Acta.

[52] Wadsö I., Goldberg R.N. (2001). Standards in isothermal microcalorimetry (IUPAC Technical Report). Pure Appl. Chem..

[53] Paŀecz B. (1995). The enthalpies of interaction of glycine with some amides and ureas in water at 25 °C. J. Solut. Chem..

[54] Desnoyers J.E., Perron G., Avedikian L., Morel J.-P. (1976). Enthalpies of the urea-tert-butanol-water system at 25 °C. J. Solut. Chem..

[55] Usacheva T.R., Kuz’Mina I.A., Sharnin V.A., Chernov I.V., Matteoli E. (2011). The influence of the composition of an aqueous-acetone solvent on the thermodynamic characteristics of complex formation of 18-crown-6 ether with glycine. Russ. J. Phys. Chem. A.

