# Supplementary material for: Composition of the Solvation Shell of the Selected Cyclic Ethers (1,4-Dioxane, 12-Crown-4, 15-Crown-5 and 18-Crown-6) in a Mixture of Formamide with Water at Four Temperatures
Source: Molecules. 2023 Feb 26;28(5):2169. doi: 10.3390/molecules28052169 (PMC10004068; doi:10.3390/molecules28052169)
Supplement: Supplementary file 1 [file molecules-28-02169-s001.zip › molecules-2256458-supplementary.pdf]

Composition of the solvation shell of the selected cyclic ethers (1,4-dioxane, 12-crown-4, 15-crown-5 and 18-crown-6) in a mixture of formamide with water at four temperature

Małgorzata Jóźwiak*[a], Monika A. Trzmielak[a], Michał Wasiak[a], Katarzyna Łudzik-Dychto[a]

[a]University of Lodz, Faculty of Chemistry, Department of Physical Chemistry, Pomorska 165, 90−236 Lodz, Poland

*corresponding author: malgorzata.jozwiak@chemia.uni.lodz.pl

Supplementary Materials

Table S1. Total number of water (W) and formamide (F) molecules in the solvation sphere of 1,4-dioxane $r = (r_W + r_F)$, mole fraction of formamide (F) in the solvation sphere of the solute (y_F), entropic factor of the transfer entropy of 1,4-dioxane in the process of preferential solvation at $T = 293.15$ K in dependency of the mole fraction of water (x_W) or formamide (x_F) in the mixture F +W.

1,4-dioxane								
formamide (F) + water (W) at $T = 293.15$ K								
x_W	r	r_W	r_F	x_F	y_F	$T\Delta_{tr}S$ / kJ·mol^{-1} $(W \rightarrow F+W)_{(B)}$	$T\Delta_{tr}S$ / kJ·mol^{-1} $(W \rightarrow F+W)_{(A)}$	δ^a
0.000	3.8	0.000	3.800	1.000	1.000	0.00	0.00	0.00
0.100	3.8	0.063	3.737	0.900	0.983	−0.53	−0.53	0.00
0.200	3.8	0.139	3.661	0.800	0.963	−1.08	−1.08	0.00
0.300	3.8	0.232	3.568	0.700	0.939	−1.65	−1.65	0.00
0.400	3.8	0.349	3.451	0.600	0.908	−2.23	−2.23	0.00
0.500	3.8	0.501	3.299	0.500	0.868	−2.81	−2.81	0.00
0.600	3.8	0.704	3.096	0.400	0.815	−3.35	−3.35	0.00
0.700	3.8	0.992	2.808	0.300	0.739	−3.79	−3.78	−0.01
0.800	3.8	1.432	2.368	0.200	0.623	−3.93	−3.93	0.00
0.900	3.8	2.189	1.611	0.100	0.424	−3.29	−3.29	0.00
0.920	3.8	2.411	1.389	0.080	0.366	−2.96	−2.96	0.00
0.940	3.8	2.670	1.130	0.060	0.297	−2.51	−2.51	0.00
0.960	3.8	2.977	0.823	0.040	0.217	−1.91	−1.91	0.00
0.980	3.8	3.347	0.453	0.020	0.119	−1.10	−1.10	0.00
1.000	3.8	3.800	0.000	0.000	0.000	0.00	0.00	0.00

[a] $\delta = T\Delta_{tr}S(W \rightarrow F+W)_{(B)} - T\Delta_{tr}S(W \rightarrow F+W)_{(A)}$

Table S2. Total number of water (W) and formamide (F) molecules in the solvation sphere of 1,4-dioxane $r = (r_W + r_F)$, mole fraction of formamide (F) in the solvation sphere of the solute (y_F), entropic factor of the transfer entropy of 1,4-dioxane in the process of preferential solvation at $T = 298.15$ K in dependency of the mole fraction of water (x_W) or formamide (x_F) in the mixture F +W.

1,4-dioxane								
formamide (F) + water (W) at $T = 298.15$ K								
x_W	r	r_W	r_F	x_F	y_F	$T\Delta_{tr}S$ / kJ·mol^{-1} $(W \rightarrow F+W)_{(B)}$	$T\Delta_{tr}S$ / kJ·mol^{-1} $(W \rightarrow F+W)_{(A)}$	δ^a
0.000	3.7	0.000	3.700	1.000	1.000	0.00	0.00	0.00
0.100	3.7	0.064	3.636	0.900	0.983	−0.51	−0.51	0.00
0.200	3.7	0.141	3.559	0.800	0.962	−1.05	−1.05	0.00
0.300	3.7	0.236	3.464	0.700	0.936	−1.59	−1.59	0.00
0.400	3.7	0.354	3.346	0.600	0.904	−2.15	−2.15	0.00
0.500	3.7	0.507	3.193	0.500	0.863	−2.69	−2.69	0.00
0.600	3.7	0.712	2.988	0.400	0.808	−3.20	−3.20	0.00
0.700	3.7	1.002	2.698	0.300	0.729	−3.58	−3.58	0.00
0.800	3.7	1.439	2.261	0.200	0.611	−3.68	−3.69	0.01
0.900	3.7	2.180	1.520	0.100	0.411	−3.03	−3.03	0.00
0.920	3.7	2.394	1.306	0.080	0.353	−2.72	−2.72	0.00
0.940	3.7	2.642	1.058	0.060	0.286	−2.29	−2.29	0.00
0.960	3.7	2.934	0.766	0.040	0.207	−1.73	−1.73	0.00
0.980	3.7	3.280	0.420	0.020	0.114	−0.99	−0.99	0.00
1.000	3.7	3.700	0.000	0.000	0.000	0.00	0.00	0.00

$^a\delta = T\Delta_{tr}S(W \rightarrow F+W)_{(B)} - T\Delta_{tr}S(W \rightarrow F+W)_{(A)}$

Table S3. Total number of water (W) and formamide (F) molecules in the solvation sphere of 1,4-dioxane r = (r_W + r_F), mole fraction of formamide (F) in the solvation sphere of the solute (y_F), entropic factor of the transfer entropy of 1,4-dioxane in the process of preferential solvation at T = 303.15 K in dependency of the mole fraction of water (x_W) or formamide (x_F) in the mixture F +W.

1,4-dioxane								
formamide (F) + water (W) at T = 303.15 K								
x_W	r	r_W	r_F	x_F	y_F	$T\Delta_{tr}S$ / kJ·mol^{-1} (W → F + W)$_{(B)}$	$T\Delta_{tr}S$ / kJ·mol^{-1} (W → F + W)$_{(A)}$	δ^a
0.000	3.6	0.000	3.600	1.000	1.000	0.00	0.00	0.00
0.100	3.6	0.062	3.538	0.900	0.983	−0.51	−0.51	0.00
0.200	3.6	0.136	3.464	0.800	0.962	−1.04	−1.04	0.00
0.300	3.6	0.227	3.373	0.700	0.937	−1.58	−1.59	0.01
0.400	3.6	0.342	3.258	0.600	0.905	−2.14	−2.14	0.00
0.500	3.6	0.490	3.110	0.500	0.864	−2.68	−2.68	0.00
0.600	3.6	0.689	2.911	0.400	0.809	−3.18	−3.18	0.00
0.700	3.6	0.970	2.630	0.300	0.731	−3.56	−3.57	0.01
0.800	3.6	1.396	2.204	0.200	0.612	−3.67	−3.67	0.00
0.900	3.6	2.117	1.483	0.100	0.412	−3.02	−3.02	0.00
0.920	3.6	2.326	1.274	0.080	0.354	−2.70	−2.70	0.00
0.940	3.6	2.568	1.032	0.060	0.287	−2.28	−2.28	0.00
0.960	3.6	2.852	0.748	0.040	0.208	−1.72	−1.73	0.01
0.980	3.6	3.190	0.410	0.020	0.114	−0.99	−0.99	0.00
1.000	3.6	3.600	0.000	0.000	0.000	0.00	0.00	0.00

$^a\delta = T\Delta_{tr}S(W \rightarrow F + W)_{(B)} - T\Delta_{tr}S(W \rightarrow F + W)_{(A)}$

Table S4. Total number of water (W) and formamide (F) molecules in the solvation sphere of 1,4-dioxane r = (r_W + r_F), mole fraction of formamide (F) in the solvation sphere of the solute (y_F), entropic factor of the transfer entropy of 1,4-dioxane in the process of preferential solvation at T = 308.15 K in dependency of the mole fraction of water (x_W) or formamide (x_F) in the mixture F +W.

1,4-dioxane								
formamide (F) + water (W) at T = 308.15 K								
x_W	r	r_W	r_F	x_F	y_F	$T\Delta_{tr}S$ / kJ·mol^{-1} (W → F + W)$_{(B)}$	$T\Delta_{tr}S$ / kJ·mol^{-1} (W → F + W)$_{(A)}$	δ^a
0.000	3.6	0.000	3.600	1.000	1.000	0.00	0.00	0.00
0.100	3.6	0.063	3.537	0.900	0.983	−0.51	−0.51	0.00
0.200	3.6	0.138	3.462	0.800	0.962	−1.05	−1.05	0.00
0.300	3.6	0.231	3.369	0.700	0.936	−1.59	−1.59	0.00
0.400	3.6	0.347	3.253	0.600	0.904	−2.15	−2.15	0.00
0.500	3.6	0.497	3.103	0.500	0.862	−2.69	−2.69	0.00
0.600	3.6	0.697	2.903	0.400	0.806	−3.20	−3.19	−0.01
0.700	3.6	0.979	2.621	0.300	0.728	−3.58	−3.58	0.01
0.800	3.6	1.405	2.195	0.200	0.610	−3.69	−3.68	−0.01
0.900	3.6	2.125	1.475	0.100	0.410	−3.03	−3.03	0.00
0.920	3.6	2.332	1.268	0.080	0.352	−2.72	−2.72	0.00
0.940	3.6	2.573	1.027	0.060	0.285	−2.29	−2.30	0.01
0.960	3.6	2.856	0.744	0.040	0.207	−1.73	−1.73	0.00
0.980	3.6	3.193	0.407	0.020	0.113	−0.99	−0.99	0.00
1.000	3.6	3.600	0.000	0.000	0.000	0.00	0.00	0.00

[a] $\delta = T\Delta_{tr}S(W \rightarrow F + W)_{(B)} - T\Delta_{tr}S(W \rightarrow F + W)_{(A)}$

Table S5. Total number of water (W) and formamide (F) molecules in the solvation sphere of 12C4 $r = (r_W + r_F)$, mole fraction of formamide (F) in the solvation sphere of the solute (y_F), entropic factor of the transfer entropy of 12C4 in the process of preferential solvation at $T = 293.15$ K in dependency of the mole fraction of water (x_W) or formamide (x_F) in the mixture F +W.

12-crown-4								
formamide (F) + water (W) at $T = 293.15$ K								
x_W	r	r_W	r_F	x_F	y_F	$T\Delta_{tr}S$ / kJ·mol^{-1} $(W \rightarrow F+W)_{(B)}$	$T\Delta_{tr}S$ / kJ·mol^{-1} $(W \rightarrow F+W)_{(A)}$	δ^a
0.000	13.1	0.000	13.100	1.000	1.000	0.00	0.00	0.00
0.100	13.1	0.353	12.747	0.900	0.973	−1.30	−1.30	0.00
0.200	13.1	0.768	12.332	0.800	0.941	−2.59	−2.59	0.00
0.300	13.1	1.264	11.836	0.700	0.904	−3.87	−3.87	0.00
0.400	13.1	1.867	11.233	0.600	0.857	−5.08	−5.08	0.00
0.500	13.1	2.614	10.486	0.500	0.800	−6.17	−6.17	0.00
0.600	13.1	3.566	9.534	0.400	0.728	−7.04	−7.04	0.00
0.700	13.1	4.819	8.281	0.300	0.632	−7.48	−7.49	0.01
0.800	13.1	6.544	6.556	0.200	0.500	−7.15	−7.14	−0.01
0.900	13.1	9.065	4.035	0.100	0.308	−5.26	−5.26	0.00
0.920	13.1	9.715	3.385	0.080	0.258	−4.57	−4.57	0.00
0.940	13.1	10.432	2.668	0.060	0.204	−3.73	−3.73	0.01
0.960	13.1	11.226	1.874	0.040	0.143	−2.71	−2.71	0.00
0.980	13.1	12.110	0.990	0.020	0.076	−1.48	−1.48	0.00
1.000	13.1	13.100	0.000	0.000	0.000	0.00	0.00	0.00

$^a\delta = T\Delta_{tr}S(W \rightarrow F + W)_{(B)} - T\Delta_{tr}S(W \rightarrow F + W)_{(A)}$

Table S6. Total number of water (W) and formamide (F) molecules in the solvation sphere of 12C4 $r = (r_W + r_F)$, mole fraction of formamide (F) in the solvation sphere of the solute (y_F), entropic factor of the transfer entropy of 12C4 in the process of preferential solvation at $T = 298.15$ K in dependency of the mole fraction of water (x_W) or formamide (x_F) in the mixture F +W.

12-crown-4								
formamide (F) + water (W) at $T = 298.15$ K								
x_W	r	r_W	r_F	x_F	y_F	$T\Delta_{tr}S$ / kJ·mol^{-1} $(W \rightarrow F+W)_{(B)}$	$T\Delta_{tr}S$ / kJ·mol^{-1} $(W \rightarrow F+W)_{(A)}$	δ^a
0.000	12.5	0.000	12.500	1.000	1.000	0.00	0.00	0.00
0.100	12.5	0.339	12.161	0.900	0.973	−1.25	−1.25	0.00
0.200	12.5	0.738	11.762	0.800	0.941	−2.50	−2.50	0.00
0.300	12.5	1.214	11.286	0.700	0.903	−3.73	−3.73	0.00
0.400	12.5	1.791	10.709	0.600	0.857	−4.90	−4.90	0.00
0.500	12.5	2.507	9.993	0.500	0.799	−5.95	−5.95	0.00
0.600	12.5	3.417	9.083	0.400	0.727	−6.78	−6.78	0.00
0.700	12.5	4.614	7.886	0.300	0.631	−7.21	−7.21	0.00
0.800	12.5	6.258	6.242	0.200	0.499	−6.89	−6.89	0.00
0.900	12.5	8.660	3.840	0.100	0.307	−5.07	−5.07	0.00
0.920	12.5	9.279	3.221	0.080	0.258	−4.40	−4.40	0.00
0.940	12.5	9.962	2.538	0.060	0.203	−3.59	−3.59	0.01
0.960	12.5	10.717	1.783	0.040	0.143	−2.61	−2.62	0.01
0.980	12.5	11.558	0.942	0.020	0.075	−1.43	−1.43	0.00
1.000	12.5	12.500	0.000	0.000	0.000	0.00	0.00	0.00

$^a\delta = T\Delta_{tr}S(W \rightarrow F+W)_{(B)} - T\Delta_{tr}S(W \rightarrow F+W)_{(A)}$

Table S7. Total number of water (W) and formamide (F) molecules in the solvation sphere of 12C4 $r = (r_W + r_F)$, mole fraction of formamide (F) in the solvation sphere of the solute (y_F), entropic factor of the transfer entropy of 12C4 in the process of preferential solvation at $T = 303.15$ K in dependency of the mole fraction of water (x_W) or formamide (x_F) in the mixture F +W.

12-crown-4								
formamide (F) + water (W) at $T = 303.15$ K								
x_W	r	r_W	r_F	x_F	y_F	$T\Delta_{tr}S$ / kJ·mol^{-1} $(W \rightarrow F+W)_{(B)}$	$T\Delta_{tr}S$ / kJ·mol^{-1} $(W \rightarrow F+W)_{(A)}$	δ^a
0.000	11.7	0.000	11.700	1.000	1.000	0.00	0.00	0.00
0.100	11.7	0.317	11.383	0.900	0.973	−1.19	−1.19	0.00
0.200	11.7	0.689	11.011	0.800	0.941	−2.38	−2.39	0.01
0.300	11.7	1.134	10.566	0.700	0.903	−3.55	−3.55	0.00
0.400	11.7	1.674	10.026	0.600	0.857	−4.67	−4.67	0.00
0.500	11.7	2.343	9.357	0.500	0.800	−5.67	−5.67	0.00
0.600	11.7	3.194	8.506	0.400	0.727	−6.47	−6.47	0.00
0.700	11.7	4.314	7.386	0.300	0.631	−6.88	−6.88	0.00
0.800	11.7	5.853	5.847	0.200	0.500	−6.57	−6.57	0.00
0.900	11.7	8.102	3.598	0.100	0.308	−4.83	−4.83	0.00
0.920	11.7	8.683	3.017	0.080	0.258	−4.20	−4.20	0.00
0.940	11.7	9.322	2.378	0.060	0.203	−3.43	−3.43	0.00
0.960	11.7	10.030	1.670	0.040	0.143	−2.49	−2.49	0.00
0.980	11.7	10.818	0.882	0.020	0.075	−1.36	−1.36	0.00
1.000	11.7	11.700	0.000	0.000	0.000	0.00	0.00	0.00

$^a\delta = T\Delta_{tr}S(W \rightarrow F+W)_{(B)} - T\Delta_{tr}S(W \rightarrow F+W)_{(A)}$

Table S8. Total number of water (W) and formamide (F) molecules in the solvation sphere of 12C4 $r = (r_W + r_F)$, mole fraction of formamide (F) in the solvation sphere of the solute (y_F), entropic factor of the transfer entropy of 12C4 in the process of preferential solvation at $T = 308.15$ K in dependency of the mole fraction of water (x_W) or formamide (x_F) in the mixture F +W.

12-crown-4								
formamide (F) + water (W) at $T = 308.15$ K								
x_W	r	r_W	r_F	x_F	y_F	$T\Delta_{tr}S$ / kJ·mol^{-1} $(W \rightarrow F+W)_{(B)}$	$T\Delta_{tr}S$ / kJ·mol^{-1} $(W \rightarrow F+W)_{(A)}$	δ^a
0.000	11.1	0.000	11.100	1.000	1.000	0.00	0.00	0.00
0.100	11.1	0.288	10.812	0.900	0.974	−1.19	−1.19	0.00
0.200	11.1	0.630	10.470	0.800	0.943	−2.38	−2.39	0.01
0.300	11.1	1.038	10.062	0.700	0.906	−3.56	−3.56	0.00
0.400	11.1	1.534	9.566	0.600	0.862	−4.70	−4.70	0.00
0.500	11.1	2.153	8.947	0.500	0.806	−5.72	−5.72	0.00
0.600	11.1	2.943	8.157	0.400	0.735	−6.55	−6.55	0.00
0.700	11.1	3.990	7.110	0.300	0.641	−7.00	−7.00	0.00
0.800	11.1	5.442	5.658	0.200	0.510	−6.74	−6.73	−0.01
0.900	11.1	7.591	3.509	0.100	0.316	−5.01	−5.01	0.00
0.920	11.1	8.151	2.949	0.080	0.266	−4.36	−4.36	0.00
0.940	11.1	8.771	2.329	0.060	0.210	−3.57	−3.57	0.00
0.960	11.1	9.460	1.640	0.040	0.148	−2.60	−2.60	0.00
0.980	11.1	10.231	0.869	0.020	0.078	−1.43	−1.43	0.00
1.000	11.1	11.100	0.000	0.000	0.000	0.00	0.00	0.00

$^a\delta = T\Delta_{tr}S(W \rightarrow F+W)_{(B)} - T\Delta_{tr}S(W \rightarrow F+W)_{(A)}$

Table S9. Total number of water (W) and formamide (F) molecules in the solvation sphere of 15C5 $r = (r_W + r_F)$, mole fraction of formamide (F) in the solvation sphere of the solute (y_F), entropic factor of the transfer entropy of 15C5 in the process of preferential solvation at $T = 293.15$ K in dependency of the mole fraction of water (x_W) or formamide (x_F) in the mixture F +W.

15-crown-5								
formamide (F) + water (W) at $T = 293.15$ K								
x_W	r	r_W	r_F	x_F	y_F	$T\Delta_{tr}S$ / kJ·mol^{-1} (W → F + W)$_{(B)}$	$T\Delta_{tr}S$ / kJ·mol^{-1} (W → F + W)$_{(A)}$	δ^a
0.000	30.3	0.000	30.300	1.000	1.000	0.00	0.00	0.00
0.100	30.3	1.165	29.135	0.900	0.962	−1.98	−1.98	0.00
0.200	30.3	2.500	27.800	0.800	0.917	−3.89	−3.89	0.00
0.300	30.3	4.048	26.252	0.700	0.866	−5.67	−5.66	−0.01
0.400	30.3	5.862	24.438	0.600	0.807	−7.24	−7.24	0.00
0.500	30.3	8.017	22.283	0.500	0.735	−8.52	−8.52	0.00
0.600	30.3	10.621	19.679	0.400	0.649	−9.33	−9.33	0.00
0.700	30.3	13.829	16.471	0.300	0.544	−9.45	−9.45	0.00
0.800	30.3	17.879	12.421	0.200	0.410	−8.46	−8.46	0.00
0.900	30.3	23.151	7.149	0.100	0.236	−5.72	−5.72	0.00
0.920	30.3	24.403	5.897	0.080	0.195	−4.86	−4.86	0.00
0.940	30.3	25.735	4.565	0.060	0.151	−3.88	−3.88	0.00
0.960	30.3	27.156	3.144	0.040	0.104	−2.76	−2.76	0.00
0.980	30.3	28.674	1.626	0.020	0.054	−1.47	−1.47	0.00
1.000	30.3	30.300	0.000	0.000	0.000	0.00	0.00	0.00

$^a\delta = T\Delta_{tr}S(W \rightarrow F + W)_{(B)} - T\Delta_{tr}S(W \rightarrow F + W)_{(A)}$

Table S10. Total number of water (W) and formamide (F) molecules in the solvation sphere of 15C5 $r = (r_W + r_F)$, mole fraction of formamide (F) in the solvation sphere of the solute (y_F), entropic factor of the transfer entropy of 15C5 in the process of preferential solvation at $T = 298.15$ K in dependency of the mole fraction of water (x_W) or formamide (x_F) in the mixture F +W.

15-crown-5								
formamide (F) + water (W) at $T = 298.15$ K								
x_W	r	r_W	r_F	x_F	y_F	$T\Delta_{tr}S$ / kJ · mol^{-1} $(W \rightarrow F + W)_{(B)}$	$T\Delta_{tr}S$ / kJ · mol^{-1} $(W \rightarrow F + W)_{(A)}$	δ^a
0.000	26.0	0.000	26.000	1.000	1.000	0.00	0.00	0.00
0.100	26.0	0.946	25.054	0.900	0.964	−1.87	−1.87	0.00
0.200	26.0	2.036	23.964	0.800	0.922	−3.68	−3.68	0.00
0.300	26.0	3.305	22.695	0.700	0.873	−5.38	−5.38	0.00
0.400	26.0	4.802	21.198	0.600	0.815	−6.91	−6.91	0.00
0.500	26.0	6.595	19.405	0.500	0.746	−8.18	−8.17	−0.01
0.600	26.0	8.779	17.221	0.400	0.662	−9.02	−9.02	0.00
0.700	26.0	11.499	14.501	0.300	0.558	−9.20	−9.20	0.00
0.800	26.0	14.981	11.019	0.200	0.424	−8.33	−8.33	0.00
0.900	26.0	19.594	6.406	0.100	0.246	−5.70	−5.70	0.00
0.920	26.0	20.703	5.297	0.080	0.204	−4.86	−4.86	0.00
0.940	26.0	21.889	4.111	0.060	0.158	−3.89	−3.89	0.00
0.960	26.0	23.161	2.839	0.040	0.109	−2.77	−2.77	0.00
0.980	26.0	24.527	1.473	0.020	0.057	−1.48	−1.48	0.00
1.000	26.0	26.000	0.000	0.000	0.000	0.00	0.00	0.00

[a] $\delta = T\Delta_{tr}S(W \rightarrow F + W)_{(B)} - T\Delta_{tr}S(W \rightarrow F + W)_{(A)}$

Table S11. Total number of water (W) and formamide (F) molecules in the solvation sphere of 15C5 $r = (r_W + r_F)$, mole fraction of formamide (F) in the solvation sphere of the solute (y_F), entropic factor of the transfer entropy of 15C5 in the process of preferential solvation at $T = 303.15$ K in dependency of the mole fraction of water (x_W) or formamide (x_F) in the mixture F +W.

15-crown-5								
formamide (F) + water (W) at $T = 303.15$ K								
x_W	r	r_W	r_F	x_F	y_F	$T\Delta_{tr}S$ / kJ·mol^{-1} $(W \rightarrow F+W)_{(B)}$	$T\Delta_{tr}S$ / kJ·mol^{-1} $(W \rightarrow F+W)_{(A)}$	δ^a
0.000	22.9	0.000	22.900	1.000	1.000	0.00	0.00	0.00
0.100	22.9	0.784	22.116	0.900	0.966	−1.81	−1.81	0.00
0.200	22.9	1.693	21.207	0.800	0.926	−3.57	−3.58	0.00
0.300	22.9	2.758	20.142	0.700	0.880	−5.24	−5.25	0.00
0.400	22.9	4.025	18.875	0.600	0.824	−6.77	−6.76	0.00
0.500	22.9	5.547	17.353	0.500	0.758	−8.05	−8.05	0.00
0.600	22.9	7.422	15.478	0.400	0.676	−8.94	−8.94	0.00
0.700	22.9	9.785	13.115	0.300	0.573	−9.20	−9.20	0.00
0.800	22.9	12.852	10.048	0.200	0.439	−8.41	−8.41	0.00
0.900	22.9	16.995	5.905	0.100	0.258	−5.84	−5.84	0.00
0.920	22.9	18.005	4.895	0.080	0.214	−5.00	−5.00	0.00
0.940	22.9	19.089	3.811	0.060	0.166	−4.02	−4.02	0.00
0.960	22.9	20.261	2.639	0.040	0.115	−2.87	−2.87	0.00
0.980	22.9	21.526	1.374	0.020	0.060	−1.54	−1.54	0.00
1.000	22.9	22.900	0.000	0.000	0.000	0.00	0.00	0.00

$^a\delta = T\Delta_{tr}S(W \rightarrow F + W)_{(B)} - T\Delta_{tr}S(W \rightarrow F + W)_{(A)}$

Table S12. Total number of water (W) and formamide (F) molecules in the solvation sphere of 15C5 $r = (r_W + r_F)$, mole fraction of formamide (F) in the solvation sphere of the solute (y_F), entropic factor of the transfer entropy of 15C5 in the process of preferential solvation at T = 308.15 K in dependency of the mole fraction of water (x_W) or formamide (x_F) in the mixture F +W.

15-crown-5								
formamide (F) + water (W) at T = 308.15 K								
x_W	r	r_W	r_F	x_F	y_F	$T\Delta_{tr}S$ / kJ · mol^{-1} $(W \rightarrow F+W)_{(B)}$	$T\Delta_{tr}S$ / kJ · mol^{-1} $(W \rightarrow F+W)_{(A)}$	δ[a]
0.000	20.3	0.000	20.300	1.000	1.000	0.00	0.00	0.00
0.100	20.3	0.654	19.646	0.900	0.968	−1.75	−1.76	0.01
0.200	20.3	1.417	18.883	0.800	0.930	−3.47	−3.47	0.00
0.300	20.3	2.315	17.985	0.700	0.886	−5.12	−5.12	0.00
0.400	20.3	3.385	16.915	0.600	0.833	−6.65	−6.64	−0.01
0.500	20.3	4.687	15.613	0.500	0.769	−7.95	−7.95	0.00
0.600	20.3	6.302	13.998	0.400	0.690	−8.89	−8.89	0.00
0.700	20.3	8.361	11.939	0.300	0.588	−9.23	−9.23	0.00
0.800	20.3	11.075	9.225	0.200	0.454	−8.54	−8.54	0.00
0.900	20.3	14.813	5.487	0.100	0.270	−6.01	−6.02	0.01
0.920	20.3	15.738	4.562	0.080	0.225	−5.17	−5.17	0.00
0.940	20.3	16.739	3.561	0.060	0.175	−4.17	−4.17	0.00
0.960	20.3	17.825	2.475	0.040	0.122	−2.99	−2.99	0.00
0.980	20.3	19.006	1.294	0.020	0.064	−1.62	−1.62	0.00
1.000	20.3	20.300	0.000	0.000	0.000	0.00	0.00	0.00

[a]$\delta = T\Delta_{tr}S(W \rightarrow F+W)_{(B)} - T\Delta_{tr}S(W \rightarrow F+W)_{(A)}$

Table S13. Total number of water (W) and formamide (F) molecules in the solvation sphere of 18C6 $r = (r_W + r_F)$, mole fraction of formamide (F) in the solvation sphere of the solute (y_F), entropic factor of the transfer entropy of 18C6 in the process of preferential solvation at $T = 293.15$ K in dependency of the mole fraction of water (x_W) or formamide (x_F) in the mixture F +W.

18-crown-6								
formamide (F) + water (W) at $T = 293.15$ K								
x_W	r	r_W	r_F	x_F	y_F	$T\Delta_{tr}S$ / kJ·mol^{-1} $(W \rightarrow F+W)_{(B)}$	$T\Delta_{tr}S$ / kJ·mol^{-1} $(W \rightarrow F+W)_{(A)}$	δ^a
0.000	61.3	0.000	61.300	1.000	1.000	0.00	0.00	0.00
0.100	61.3	2.855	58.445	0.900	0.953	−2.89	−2.90	0.01
0.200	61.3	6.076	55.224	0.800	0.901	−5.59	−5.59	0.00
0.300	61.3	9.726	51.574	0.700	0.841	−8.02	−8.02	0.00
0.400	61.3	13.902	47.398	0.600	0.773	−10.07	−10.07	0.00
0.500	61.3	18.731	42.569	0.500	0.694	−11.60	−11.60	0.00
0.600	61.3	24.373	36.927	0.400	0.602	−12.41	−12.41	0.00
0.700	61.3	31.051	30.249	0.300	0.493	−12.21	−12.21	0.00
0.800	61.3	39.089	22.211	0.200	0.362	−10.56	−10.56	0.00
0.900	61.3	48.941	12.359	0.100	0.202	−6.83	−6.83	0.00
0.920	61.3	51.183	10.117	0.080	0.165	−5.76	−5.76	0.00
0.940	61.3	53.533	7.767	0.060	0.127	−4.55	−4.55	0.00
0.960	61.3	55.996	5.304	0.040	0.087	−3.19	−3.19	0.00
0.980	61.3	58.581	2.719	0.020	0.044	−1.68	−1.69	0.01
1.000	61.3	61.300	0.000	0.000	0.000	0.00	0.00	0.00

$^a\delta = T\Delta_{tr}S(W \rightarrow F + W)_{(B)} - T\Delta_{tr}S(W \rightarrow F + W)_{(A)}$

Table S14. Total number of water (W) and formamide (F) molecules in the solvation sphere of 18C6 $r = (r_W + r_F)$, mole fraction of formamide (F) in the solvation sphere of the solute (y_F), entropic factor of the transfer entropy of 18C6 in the process of preferential solvation at $T = 298.15$ K in dependency of the mole fraction of water (x_W) or formamide (x_F) in the mixture F +W.

18-crown-6								
formamide (F) + water (W) at $T = 298.15$ K								
x_W	r	r_W	r_F	x_F	y_F	$T\Delta_{tr}S$ / kJ·mol^{-1} $(W \rightarrow F+W)_{(B)}$	$T\Delta_{tr}S$ / kJ·mol^{-1} $(W \rightarrow F+W)_{(A)}$	δ^a
0.000	59.5	0.000	59.500	1.000	1.000	0.00	0.00	0.00
0.100	59.5	2.711	56.789	0.900	0.954	−2.98	−2.98	0.00
0.200	59.5	5.776	53.724	0.800	0.903	−5.77	−5.77	0.00
0.300	59.5	9.256	50.244	0.700	0.844	−8.29	−8.30	0.01
0.400	59.5	13.252	46.248	0.600	0.777	−10.44	−10.44	0.00
0.500	59.5	17.891	41.609	0.500	0.699	−12.05	−12.05	0.00
0.600	59.5	23.329	36.171	0.400	0.608	−12.93	−12.93	0.00
0.700	59.5	29.795	29.705	0.300	0.499	−12.76	−12.76	0.00
0.800	59.5	37.620	21.880	0.200	0.368	−11.08	−11.09	0.01
0.900	59.5	47.281	12.219	0.100	0.205	−7.20	−7.20	0.00
0.920	59.5	49.488	10.012	0.080	0.168	−6.08	−6.08	0.00
0.940	59.5	51.807	7.693	0.060	0.129	−4.80	−4.81	0.01
0.960	59.5	54.243	5.257	0.040	0.088	−3.38	−3.38	0.00
0.980	59.5	56.805	2.695	0.020	0.045	−1.78	−1.78	0.00
1.000	59.5	59.500	0.000	0.000	0.000	0.00	0.00	0.00

$^a\delta = T\Delta_{tr}S(W \rightarrow F+W)_{(B)} - T\Delta_{tr}S(W \rightarrow F+W)_{(A)}$

Table S15. Total number of water (W) and formamide (F) molecules in the solvation sphere of 18C6 $r = (r_W + r_F)$, mole fraction of formamide (F) in the solvation sphere of the solute (y_F), entropic factor of the transfer entropy of 18C6 in the process of preferential solvation at $T = 303.15$ K in dependency of the mole fraction of water (x_W) or formamide (x_F) in the mixture F +W.

18-crown-6								
formamide (F) + water (W) at $T = 303.15$ K								
x_W	r	r_W	r_F	x_F	y_F	$T\Delta_{tr}S$ / kJ·mol^{-1} (W → F + W)$_{(B)}$	$T\Delta_{tr}S$ / kJ·mol^{-1} (W → F + W)$_{(A)}$	δ^a
0.000	57.6	0.000	57.600	1.000	1.000	0.00	0.00	0.00
0.100	57.6	2.629	54.971	0.900	0.954	−2.93	−2.93	0.00
0.200	57.6	5.591	52.009	0.800	0.903	−5.68	−5.68	0.00
0.300	57.6	8.963	48.637	0.700	0.844	−8.16	−8.16	0.00
0.400	57.6	12.832	44.768	0.600	0.777	−10.27	−10.27	0.00
0.500	57.6	17.321	40.279	0.500	0.699	−11.86	−11.86	0.00
0.600	57.6	22.587	35.013	0.400	0.608	−12.72	−12.72	0.00
0.700	57.6	28.848	28.752	0.300	0.499	−12.55	−12.55	0.00
0.800	57.6	36.424	21.176	0.200	0.368	−10.90	−10.90	0.00
0.900	57.6	45.775	11.825	0.100	0.205	−7.09	−7.08	−0.01
0.920	57.6	47.911	9.689	0.080	0.168	−5.98	−5.98	0.00
0.940	57.6	50.155	7.445	0.060	0.129	−4.73	−4.73	0.00
0.960	57.6	52.513	5.087	0.040	0.088	−3.32	−3.32	0.00
0.980	57.6	54.991	2.609	0.020	0.045	−1.75	−1.75	0.00
1.000	57.6	57.600	0.000	0.000	0.000	0.00	0.00	0.00

$^a\delta = T\Delta_{tr}S(\mathrm{W} \rightarrow \mathrm{F} + \mathrm{W})_{(B)} - T\Delta_{tr}S(\mathrm{W} \rightarrow \mathrm{F} + \mathrm{W})_{(A)}$

Table S16. Total number of water (W) and formamide (F) molecules in the solvation sphere of 18C6 $r = (r_W + r_F)$, mole fraction of formamide (F) in the solvation sphere of the solute (y_F), entropic factor of the transfer entropy of 18C6 in the process of preferential solvation at $T = 308.15$ K in dependency of the mole fraction of water (x_W) or formamide (x_F) in the mixture F +W.

18-crown-6								
formamide (F) + water (W) at $T = 308.15$ K								
x_W	r	r_W	r_F	x_F	y_F	$T\Delta_{tr}S$ / kJ·mol^{-1} (W → F + W)$_{(B)}$	$T\Delta_{tr}S$ / kJ·mol^{-1} (W → F + W)$_{(A)}$	δ[a]
0.000	55.9	0.000	55.900	1.000	1.000	0.00	0.00	0.00
0.100	55.9	2.491	53.409	0.900	0.955	−3.02	−3.02	0.00
0.200	55.9	5.311	50.589	0.800	0.905	−5.85	−5.85	0.00
0.300	55.9	8.525	47.375	0.700	0.847	−8.43	−8.43	0.00
0.400	55.9	12.228	43.672	0.600	0.781	−10.63	−10.63	0.00
0.500	55.9	16.531	39.369	0.500	0.704	−12.31	−12.31	0.00
0.600	55.9	21.603	34.297	0.400	0.614	−13.24	−13.24	0.00
0.700	55.9	27.666	28.234	0.300	0.505	−13.11	−13.11	0.00
0.800	55.9	35.041	20.859	0.200	0.373	−11.43	−11.43	0.00
0.900	55.9	44.202	11.698	0.100	0.209	−7.47	−7.47	0.00
0.920	55.9	46.311	9.589	0.080	0.172	−6.31	−6.30	−0.01
0.940	55.9	48.525	7.375	0.060	0.132	−4.99	−4.99	0.00
0.960	55.9	50.853	5.047	0.040	0.090	−3.51	−3.52	0.01
0.980	55.9	53.311	2.589	0.020	0.046	−1.86	−1.85	−0.01
1.000	55.9	55.900	0.000	0.000	0.000	0.00	0.00	0.00

[a] $\delta = T\Delta_{tr}S(W \rightarrow F + W)_{(B)} - T\Delta_{tr}S(W \rightarrow F + W)_{(A)}$
